# Elucidating Black α-CsPbI_3_ Perovskite Stabilization via PPD Bication-Conjugated Molecule Surface Passivation: Ab Initio Simulations

**DOI:** 10.1021/acsami.4c05092

**Published:** 2024-07-18

**Authors:** José E. González, João G. Danelon, Juarez L. F. Da Silva, Matheus P. Lima

**Affiliations:** † São Carlos Institute of Chemistry, University of São Paulo, P.O. Box 780, 13560-970 São Carlos, SP, Brazil; ‡ Department of Physics, Federal University of São Carlos, 13565-905 São Carlos, SP, Brazil

**Keywords:** perovskites, CsPbI_3_, surface passivation, stability, PPD (*p*-phenylenediamine), black phase, density functional theory

## Abstract

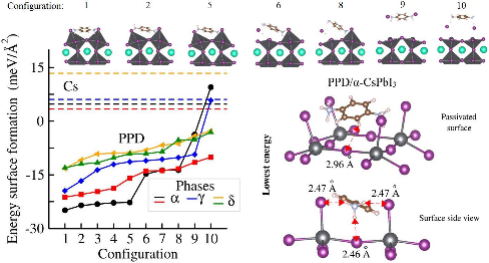

The cubic α-CsPbI_3_ phase stands out as one of the most promising perovskite compounds for solar cell applications due to its suitable electronic band gap of 1.7 eV. However, it exhibits structural instability under operational conditions, often transforming into the hexagonal non-perovskite δ-CsPbI_3_ phase, which is unsuitable for solar cell applications because of the large band gap (e.g., ∼2.9 eV). Thus, there is growing interest in identifying possible mechanisms for increasing the stability of the cubic α-CsPbI_3_ phase. Here, we report a theoretical investigation, based on density functional theory calculations, of the surface passivation of the α-, γ-, and δ-CsPbI_3_(100) surfaces using the C_6_H_4_(NH_3_)_2_ [*p*-phenylenediamine (PPD)] and Cs species as passivation agents. Our calculations and analyses corroborate recent experimental findings, showing that PPD passivation effectively stabilizes the cubic α-CsPbI_3_ perovskite against the cubic-to-hexagonal phase transition. The PPD molecule exhibits covalent-dominating bonds with the substrate, which makes it more resistant to distortion than the ionic bonds dominant in perovskite bulks. By contrasting these results with the natural Cs passivation, we highlight the superior stability of the PPD passivation, as evidenced by the negative surface formation energies, unlike the positive values observed for the Cs passivation. This disparity is due to the covalent characteristics of the molecule/surface interaction of PPD, as opposed to the purely ionic interaction seen with the Cs passivation. Notably, the PPD passivation maintains the optoelectronic properties of the perovskites because the electronic states derived from the PPD molecules are localized far from the band gap region, which is crucial for optoelectronic applications.

## 1 Introduction

In approximately two decades, the power conversion efficiency (PCE) of halide perovskite solar cells has reached 25.5%,^1^ firmly establishing their presence in the competitive landscape of solar energy materials.^2^ Independent research groups have obtained similar PCE values,^3,4^ and therefore these results have played a crucial role in increasing interest in perovskite-based materials,^5,6^ in particular, in the study of organic–inorganic halide perovskites.^7,8^ However, great success in efficiency improvement still faces structural instabilities in operating conditions triggered by interaction with ambient molecules,^9,10^ heat,^11,12^ and light.^13^ For example, reactions involving PbI_2_, CH_3_NH_3_I and H_2_O promote irreversible degradation,^14^ which reduces PCE over time.

The cubic polymorph α-CsPbI_3_ (also called black phase) emerges as a highly promising candidate for photovoltaic applications, attributed to its optimal electronic band gap of 1.73 eV,^15^ cost-effective fabrication process,^16^ and superior absorption coefficient.^17,18^ The power conversion efficiency (PCE) record for α-CsPbI_3_ based solar cells stands at approximately 21%,^19^ although a multitude of PCE values has been reported, including 17.1%,^20^ 14.1%,^21^ and 13.1%.^22^ Nonetheless, the α-CsPbI_3_ suffers from structural instabilities,^23^ as it is stable only at temperatures exceeding 300 °C.^24,25^

At room temperature, the most stable CsPbI_3_ phase is the undesirable non-perovskite hexagonal δ-phase (also called yellow phase) and it has a band gap of about 2.95 eV,^26^ which prevents applications in solar cells. Recently, a different phase has been identified at room temperature, which has an orthorhombic structure (γ-phase) instead of the cubic α-phase.^27^ It has an intermediary instability, with formation energy between the α- and δ-phases. Thus, the large number of polymorph structures introduce challenges for real-life applications based on the compound CsPbI_3_. Therefore, there is great interest in improving our understanding of the instabilities of the CsPbI_3_ phases and the identification of strategies to stabilize the α-phase at room temperature, which is a promising phase for photovoltaic applications.

Researchers have proposed several methods to stabilize the α-CsPbI_3_ phase compared to the non-perovskite δ-CsPbI_3_ phase at room temperature. For example, substitution of Pb^2+^ by bivalent cations such as Ba^2+^, Sr^2+^, Sn^2+^ and Mn^2+^ can enhance the stability of the α-phase.^28^ Moreover, Cs atoms are too small to prevent tilting of the PbI_6_ octahedra, favoring the δ-CsPbI_3_ phase. Therefore, the formation of alloys that alter the concentration of Cs by mixing them with organic cations such as formamidinium and methylammonium adjusts the stability of the α-phase.^29,30^ Application of pressure also stabilizes the α-CsPbI_3_ phase, and a few experiments have demonstrated that heating and rapidly cooling the δ-CsPbI_3_ phase under pressure preserves the α-CsPbI_3_ phase.^31,32^

In this context, experiments have also demonstrated improved structural stability in perovskites through surface passivation. For example, in CH_3_NH_3_PbI_3_ perovskites, replacing the surface cations methylammonium (CH_3_NH_3_^+^, MA) with tetra-methylammonium (TMA) and tetra-ethylammonium (TEA) has contributed to protect against moisture stability.^33^ Similarly, in α-CsPbI_3_ perovskites, replacing the surface metallic cation Cs with C_6_H_4_(NH_3_)_2_ (p-phenylenediamine, PPD) molecules^34^ has opened a new pathway to stabilize perovskites in their black phases using organic molecules. However, despite achieving these phase stability tunings, clear and unambiguous demonstrations of the mechanisms behind the phase stability of α-CsPbI_3_ are not evident, especially from an atomistic quantum theory point of view in the surface passivation process, which has attracted the attention of the community in recent years.^35,36^

Thus, in this work, we performed a theoretical investigation of the passivation of low Miller index α-, γ-, and δ-CsPbI_3_ surfaces with PPD molecules. To achieve this, we initially simulated these perovskites in their bulk structure, ensuring that the structural and electrical properties of our optimized structures closely matched the average values of experimental results reported in the literature. Furthermore, we conducted a study of PPD molecules in their gas phase, revealing their propensity to bond with iodine atoms and form PPDI molecules. Finally, in the surface passivation process, we found an energetic preferential configuration of the PPD molecule with an aromatic ring C_6_ almost parallel to the surface of the perovskite, and a lowest surface energy for PPD saturating the α-CsPbI_3_ phase, corroborating experimental works.^34^ Furthermore, we proceed with computational characterization of structural, electronic and optical properties.

## 2 Theoretical Approach and Computational Details

### 2.1 Total Energy Calculations

Our total energy calculations were based on the density functional theory (DFT) framework^37,38^ within the formulation proposed by Perdew–Burke–Ernzerhof (PBE)^39^ for the exchange-correlation energy functional. However, it is well-known that plain DFT-PBE does not provide an accurate description of long-range van der Waals (vdW) interactions,^40,41^ and hence we used the D3 vdW corrections proposed by Grimme^42^ to ensure a better description of the interactions between molecules and surfaces. Furthermore, to improve the description of the electronic band structures, we used the hybrid functional proposed by Heyd–Scuseria–Ernzerhof (HSE),^43^ where the percentage of the nonlocal Fock contribution (α_*XX*_) was tuned to reproduce the fundamental experimental band gap for the pristine CsPbI_3_ bulk phases.^26,44−46^ The all-electron full-potential projector augmented wave (PAW) method^47,48^ solves the Kohn–Sham (KS) equations^38^ as implemented in the Vienna *ab initio* simulation package (VASP),^48,49^ version 5.4.4, where the KS orbitals are expanded by plane waves.

The equilibrium structures of the three-dimensional (3D) bulk perovskites were obtained by optimizing the stress tensor and atomic forces using a plane wave cutoff energy of 634 eV, which is twice the largest recommended cutoff energy (ENMAX*max*) among the PAW projectors selected for the bulk species (Pb, Cs and I). We used such a higher cutoff energy to reduce errors originating from Pulay stress.^30,50^ All remaining bulk properties evaluated from frozen structure calculations adopt a smaller cutoff energy of 356.84 eV, which corresponds to 1.125 × *ENMAXmax*, that is, 12.5% higher than the recommended maximum value. For the integration of the Brillouin zone, we sampled the reciprocal space with equivalent **k**-meshes for all structures using the automatic generation procedured implemented within VASP with a parameter *R*_*k*_ of 25 Å. It produce **k**-meshes of 4 × 4 × 4, 3 × 5 × 2, and 5 × 2 × 1 for the α-, γ-, and δ-CsPbI_3_ bulk unit cells, respectively.

For surface calculations (i.e., surface passivation studies), we optimized only the atomic positions; that is, the equilibrium lattice parameters are not optimized. We adopted a plane wave cutoff energy of 473.51 eV (1.125 × *ENMAX*_*max*_) due to the presence of the N atoms, which requires higher cutoff energies. The integration of the Brillouin zones were done using a **k**-mesh sample defined by *R*_*k*_ = 25 Å, that is, 2 × 4 × 1 and 3 × 3 × 1 for the surface units 2 × 1 and  in phase α, respectively; 3 × 3 × 1 for the surface unit cell in the γ-phase; and 3 × 2 × 1 for the surface unit cell (1 × 1) in the δ-phase. The equilibrium configurations were obtained once the atomic forces in each atom is lower than 25 meV/Å using an energy tolerance criterion of 1 × 10^–5^ eV to break the self-consistent field KS cycle.

### 2.2 Molecular Structural Models

The following sections describe the selected bulk CsPbI_3_ structures, the slab model for each phase, and details on initial configurations for cationic surface passivation.

#### 2.2.1 Bulk CsPbI_3_ Phases

This work considers three bulk phases for the CsPbI_3_ compound: (*i*) the α-CsPbI_3_ phase, which has an ideal cubic structure with space group Pm3̅n and a primitive unit cell containing five atoms, one Cs atom in the center, one Pb atom at the cubic vertex, and three I atoms in the middle of the edges;^51−53^ (*ii*) the γ-CsPbI_3_ phase, constructed from a  supercell of the α phase with octahedral distortions and in-phase rotations along the [100] direction, belonging to space group *Pnma* with a unit cell of four Cs, four Pb, and 12 I atoms;^51−53^ (*iii*) the δ-CsPbI_3_ phase, a non-perovskite structure also with space group *Pnma* and an orthorhombic unit cell containing 4 Cs, 4 Pb, and 12 I atoms, where PbI_6_ octahedra share their edges rather than corners as in the α- and γ-phases.^51,53−55^ The α- and γ-phases are known as black phases, while the δ-phase is described as yellow.

#### 2.2.2 Unpassivated CsPbI_3_(100) Surfaces

**Figure 1:**
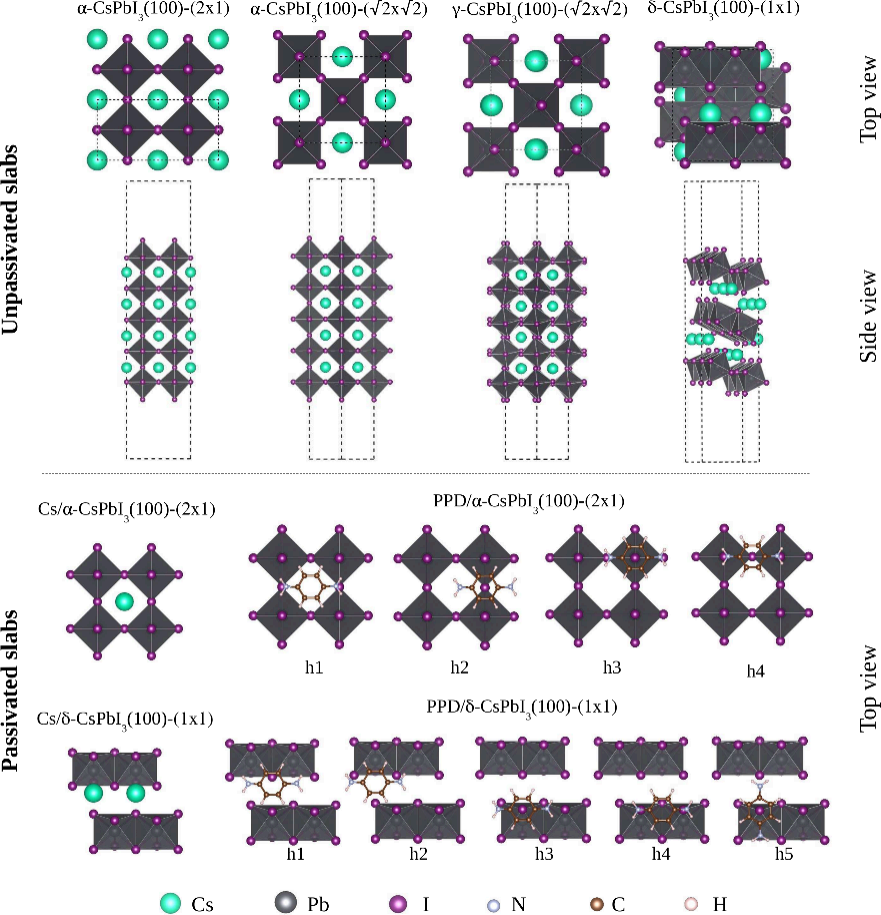
Top and side views of unpassivated perovskites (top panel). Initial adsorption configurations for surface passivation with the Cs and PPD species (bottom panel).

To investigate surface effects, we used slab geometries with 5 octahedral layers and (100) surface terminations for the α- and γ-phases, and 3 octahedral layers for the yellow phase, ensuring similar thickness for all slabs. We select CsI termination for its lowest surface energy.^56^ The lateral lattice parameters are fixed at their DFT-PBE+D3 bulk values, with a vacuum thickness of 15 Å between the periodic slabs. The top panel of Figure 1 shows views of the unpassivated surfaces. Different unit cells of the surface are considered for passivation: 2 × 1 and  for α-CsPbI_3_;  for γ-CsPbI_3_; and 1 × 1 for δ-CsPbI_3_. The unpassivated slabs of the black phases (Cs_8_Pb_10_I_32_) and the yellow phase (Cs_8_Pb_12_I_36_) result in a total oxidation state of −4 and maintain charge neutrality with four additional electrons, achieved by adding two Cs atoms to our one PPD molecule per surface.

#### 2.2.3 Passivation of the CsPbI_3_(100) Surfaces

We constructed PPD and Cs passivated surfaces by adding one PPD molecule or two Cs atoms to the top and bottom of each unpassivated slab. The systems are denoted as *x*/α-CsPbI_3_(100)-(2 × 1), *x*/α-CsPbI_3_(100)-, *x*/γ-CsPbI_3_(100)-, and *x*/δ-CsPbI_3_(100)-(1 × 1), where *x* = PPD or Cs.

For black-phase passivation, we used four orientations for the PPD molecules, all horizontal to the surface: *h*_1_ (with NH_3_ groups above removed Cs atoms), *h*_2_ (center of the C_6_ ring above removed Cs atom), *h*_3_ (with NH_3_ groups above surface iodine atoms) and *h*_4_ (center of the C_6_ ring above surface iodine atoms). In addition, six random configurations (*r*_1_ to *r*_6_) were considered, with PPD nearly parallel to the surface. For the yellow phase, we considered five horizontal symmetric configurations (*h*_1_–*h*_5_) and five additional random configurations, as shown in the lower panel of Figure 1. The initial vertical distance between PPD molecules and surfaces was set to 2.5 Å.

To optimize geometries, we preserved the bulk nature in the middle of the slabs by freezing the central octahedra layer at their bulk values while allowing other atoms to move under conjugate gradient force optimization. This procedure ensures that geometry optimizations maintain point-inversion symmetry throughout the entire process, with advantages such as (*i*) ensuring that optimized configurations have equivalent passivated surfaces, a requirement for evaluating well-defined surface energies and passivator-dependent optoelectronic properties; (*ii*) avoiding the need for dipole corrections; and (*iii*) expediting the calculation time.

## 3 Results and Discussion

To analyze the surface passivation of CsPbI_3_ perovskites using the PPD molecule and the Cs atoms, we organized the discussion into three parts, namely, bulk CsPbI_3_ perovskites (Section 3.1), PPD molecules in gas phase (Section 3.2), and passivated surfaces (Sections 3.3–3.8). (Sections 3.4-3.8).

### 3.1 Bulk CsPbI_3_ Perovskites

Here, we analyzed selected physical-chemical properties of the α-, γ-, and δ-CsPbI_3_ bulk perovskites. Table 1 shows some energetic and structural descriptors of these systems, namely the calculated lattice constants (*a*_0_, *b*_0_, and *c*_0_), average effective coordination number in Pb atom (*ECN*_*av*_^Pb^), average angles Pb– I– Pb (θ_*av*_^PbIPb^), average distance between Pb and I (*d*_*av*_^PbI^), and relative (Δ*E*_*tot*_), formation (*E*_*F*_), and cohesive (*E*_*coh*_) energies per atom. These properties were calculated using the PBE+D3 method. Additionally, Table 1 also provides several experimental and theoretical values of these descriptors reported in the literature.

**Table 1:** Equilibrium Lattice Parameters and Energies of the α, γ, and δ Phases of CsPbI Bulk Perovskites^a^

method	phase	*a*_0_ (Å)	*b*_0_ (Å)	*c*_0_ (Å)	*ECN*_*av*_^Pb^	θ_*av*_^PbIPb^ (deg)	*d*_*av*_^PbI^ (Å)	Δ*E*_*tot*_ (meV)	*E*_*F*_ (meV)	*E*_*coh*_ (eV)	ref
PBE+D3	α	6.32	6.32	6.32	6.00	180.00	3.16	32.26	–19.49	–2.77	this work
Exp./PBE	α	6.29	6.29	6.29	–	–	–	–	16.40	–	(27)
PBE	α	6.39	6.39	6.39	–	–	–	–	–0.40	–	(53)
PBE	α	6.40	6.40	6.40	–	180.00	–	–	–	–	(51)
PBE	α	6.32	6.32	6.32	–	–	–	11.10	0.32	–	(52)
Exp.	α	6.30	6.30	6.30	6.00	180.00	3.15	–	–	–	(57)
LDA	α	6.15	6.15	6.15	–	–	–	0.00	–	–	(57)
Exp.	α	6.29	6.29	6.29	–	–	–	–	–29.30	–	(58)
PBE	α	6.41	6.41	6.41	–	–	3.21	–	–	–4.17	(59)
Exp.	α	6.22	6.22	6.22	–	–	–	–	–	–	(60)
average	α	6.31	6.31	6.31	6.00	180.00	3.18	–	–3.25	–4.17	–
PBE+D3	γ	9.03	8.78	12.44	6.00	161.30	3.18	20.83	–30.91	–2.78	this work
Exp./PBE	γ	8.86	8.58	12.47	5.98	154.11	3.18	–	–7.20	–	(27)
PBE	γ	9.10	8.73	12.64	–	–	–	–	–22.00	–	(53)
PBE	γ	9.13	8.66	12.64	–	154.74	–	–	–	–	(51)
PBE	γ	8.69	9.10	12.62	6.00	151.55	3.25	10.00	–	–	(54)
Exp.	γ	8.86	8.58	12.48	6.00	154.61	3.18	–	–	–	(61)
PBE	γ	8.86	8.58	12.47	–	–	–	0.00	–10.85	–	(52)
Exp.	γ	8.85	8.62	12.50	6.00	155.52	3.17	–	–	–	(57)
LDA	γ	8.96	7.93	12.22	–	–	–	44.00	–	–	(57)
average	γ	8.91	8.60	12.51	6.00	154.11	3.20	–	–13.35	–	(57)
PBE+D3	δ	4.83	10.64	18.07	5.79	93.20	3.27	0.00	–51.74	–2.80	this work
Exp./PBE	δ	4.80	10.46	17.77	–	–	–	–	–18.80	–	(27)
PBE	δ	4.88	10.89	18.21	–	–	–	–	–32.00	–	(53)
PBE	δ	4.89	10.79	18.21	–	95.09	–	–	–	–	(51)
PBE	δ	4.88	10.82	18.16	5.75	93.36	3.28	0.00	–	–	(54)
Exp.	δ	4.80	10.45	17.76	5.69	92.50	3.24	–	–	–	(61)
Exp.	δ	4.79	10.43	17.76	–	–	–	–	–175.46	–	(58)
Exp.	δ	4.60	10.40	17.90	–	–	–	–	–	–	(26)
average	δ	4.81	10.61	17.97	5.72	93.65	3.26	–	–75.46	–	–

a Lattice constants (*a*_0_, *b*_0_, *c*_0_), average effective coordination numbers on the Pb atom (*ECN*_*av*_^Pb^), average angles Pb– I– Pb (θ_*av*_^PbIPb^), average distances between Pb and I (*d*_*av*_^PbI^), and relative (Δ*E*_*tot*_), formation (*E*_*F*_), and cohesive energies per atom (*E*_*coh*_). The experimental results (Exp.) are also indicated. The average values do not include the values calculated in the work presented here.

To validate our relaxed bulk structures, we compared the calculated equilibrium lattice constants with the average values from Table 1. For the α-phase, which has a cubic cell, our calculated lattice constant is in good agreement with the average value, showing a difference of 0.16%. However, for the γ-phase, the calculated values *a*_0_ and *b*_0_ are slightly higher than the mean values of 1.30% and 2.12%, respectively, while *c*_0_ is 0.52% smaller. In the case of the δ-phase, our calculated *a*_0_, *b*_0_, and *c*_0_ are all slightly larger, showing increases of 0.51%, 0.32%, and 0.57%, respectively.

Assessment of deformations in PbI_6_ octahedra can be used to characterize the properties of bulk phases using the *ECN*_*av*_^Pb^ and θ_*av*_^PbIPb^ descriptors. As expected, we did not find any octahedral distortions in the α-phase, consistent with the literature data shown in Table 1. However, the γ-phase inherently exhibits tilting in its octahedra. Our calculated value for θ_*av*_^PbIPb^ is 4.67° higher than the average value. Thus, the distortions for the black phases increase from α-CsPbI_3_ to γ-CsPbI_3_. This contributes to the greater stability of the γ-phase over the α-phase.^57^ We obtained a reduction in the relative total energy of 11.43 meV/atom between these phases, making γ-CsPbI_3_ the lowest energy CsPbI_3_ with a black phase. This result aligns well with a difference of 11.10 meV/atom found between these phases by Falda et al.^52^ Furthermore, our results suggest that the non-perovskite δ-CsPbI_3_ phase is even more energetically stable than the γ-CsPbI_3_ phase by 20.83 meV/atom, consistent with the difference of 10.00 meV/atom reported by Chen et al.^54^

To perform a more in-depth analysis of the energetic stability of bulk perovskites, we calculated the formation energy (*E*_*F*_), which considers the possibility that bulk perovskites are built from their binary precursor compounds CsI and PbI_2_. This value was obtained by
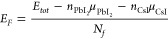
1where *E*_*tot*_ is the total energy for a given structure, *n*_PbI_2__ and *n*_CsI_ are the numbers of CsI and PbI_2_ units in the structure, respectively, and *N*_*f*_ is the number of formula units in the unit cell. μ_PbI_2__ and μ_CsI_ are the chemical potentials of CsI and PbI_2_, and these values were calculated as the total energy per unit cell of the bulk CsI and PbI_2_, respectively, representing CsI- and PbI_2_-rich conditions. The bulk CsI crystallizes in a cubic rock-salt structure, where each cesium ion is surrounded by 8 iodide ions (and vice versa), while PbI_2_ consists of closely packed hexagonal surfaces of iodine and lead ions. The iodine ions form a hexagonal lattice, and the lead ions are located between the iodine surfaces. Negative values of *E*_*F*_ indicate better energetic stability of the bulks compared to their binary constituents.

Here, we confirm the highest stability of the δ-CsPbI_3_ phase, followed by the γ-CsPbI_3_ phase in comparison with the α-CsPbI_3_ phase, based on their *E*_*F*_ values, shown in Table 1. However, the *E*_*F*_ values reported in the literature differ from each other, ranging from 16.4 to −29.3, – 7.2 to −10.85, and 18.8 to −175.46 meV for the α-, γ-, and δ-phases, respectively. However, all reports follow the same tendency as mentioned above. Thus, our results support the fact that the α-CsPbI_3_ phase is stable at high temperatures but is metastable under ambient conditions, giving rise to a phase transition to the distorted γ-CsPbI_3_ phase, which, in turn, becomes the δ-CsPbI_3_ non-perovskite phase.^54^

Another descriptor to analyze the energies stability of the bulk perovskites is the *E*_*coh*_, which is calculated as follows,
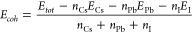
2where *n*_*i*_ is the number of atoms of the component *i* in the unitary cell (*i* = Cs, Pb and I) and *E*_*i*_ is the total energy of *i* considering the isolated atoms that form them. We obtain the same tendency as in the *E*_*F*_, the non-perovskite phase has the most negative *E*_*coh*_, followed by the γ and finally the α phase. Our results support the black-to-yellow phase transition reported so far.^62^

The values of the electronic band gaps of CsPbI_3_ are crucial for their application as a photovoltaic material. However, experimental studies report values with several discrepancies. In contrast, the electronic band gaps calculated using the PBE functional generally lead to underestimated values in comparison with the experimental references.^52−54,59^

To address this concern and precisely reproduce the experimental average band gap values (*E*_*g*_^*Exp*.^) we employed the following methodology: (*i*) Initially, we determined the band gap values using the PBE+D3 method (*E*_*g*_^PBE+D3^); (*ii*) subsequently, we accounted for the band gap reduction due to the spin–orbit coupling (SOC) effect (χ^SOC^=*E*_*g*_^PBE+D3+SOC^-*E*_*g*_^PBE+D3^); (*iii*) we calculate the rigid shift of band gaps by utilizing the hybrid functional HSE (χ^HSE^=*E*_*g*_^HSE^-*E*_*g*_^PBE+D3^),^63^ adjusting linearly the specific fraction of Hartree–Fock exchange (α_*XX*_) for each material, see Tables S6–S8 of SI file; (*iv*) finally, we applied a scissor operator technique to achieve the final corrected band gap,^30,36,64^ which is given by *E*_*g*_=*E*_*g*_^PBE+D3^+χ^SOC^+χ^HSE^.^30,36,64^ Additionally, for the end of comparison, we have calculated the rigid shifts of the band gaps by considering the α_*XX*_ = 0.25, which correspond to the usual hybrid functional HSE06.

Table 2 presents the calculated and average experimental electronic band gaps for α-, γ-, and δ-CsPbI_3_ perovskites. The hybrid HSE06 functional is not sufficient to reproduce the experimental values because the use of a mixing coefficient α_*XX*_ = 0.25 to estimate χ^HSE^ results in calculated *E*_*g*_ values narrower than their corresponding experimental values (*E*_*g*_^*Exp*.^) for all phases. However, the freedom to vary α_*XX*_ allows the simulations to exactly match the same value of the band gaps *E*_*g*_ as the average of experimental references *E*_*g*_^*Exp*.^, indicating consistency between our bulk structures and experimental measures.

**Table 2:** Electronic Band Gaps (*E*_*g*_) for the α-, γ-, and δ-CsPbI_3_ Phases Calculated with Different Approximations [PBE+D3, PBE+D3+SOC, the scissor operator (*E*_*g*_ = *E*_*g*_^PBE+D3^ + χ^SOC^ + χ^HSE^), and experimental result (*E*_*g*_^*Exp*.^)]^a^

method	α	γ	δ
*E*_*g*_^PBE+D3^	1.37	1.54	2.56
*E*_*g*_^PBE+D3+SOC^	0.37	0.65	2.08
χ^SOC^	–1.00	–0.89	–0.48
χ^HSE^	1.36	1.09	0.76
α_*XX*_	0.64	0.52	0.26
*E*_*g*_	1.73	1.74	2.85
*E*_*g*_ (α_*XX*_ = 0.25)	0.86	1.15	2.80
*E*_*g*_^*Exp*.^	1.73	1.74	2.85

a All values are presented in electronvolts, except for α^*XX*^, which has no units.

### 3.2 PPD Molecules in the Gas Phase

**Figure 2:**
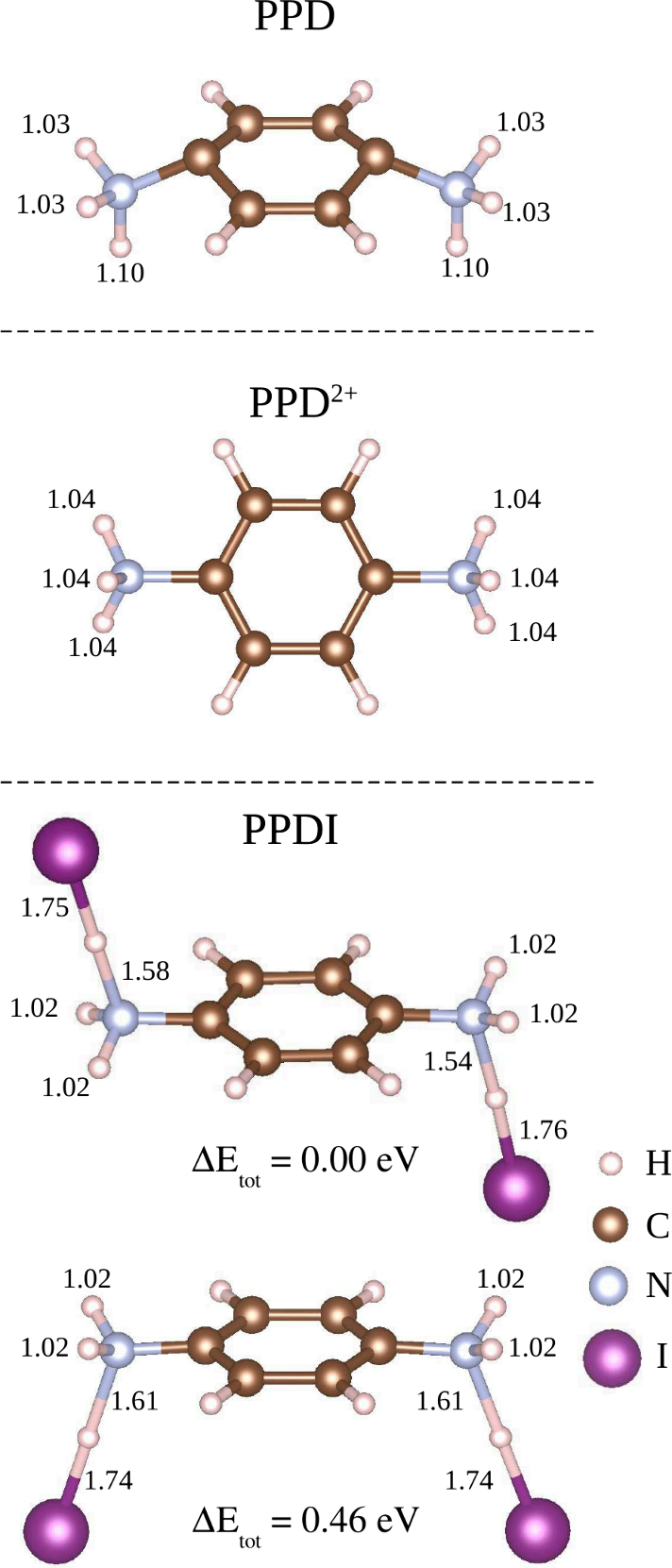
Ball and stick model for neutral PPD, PPD^2+^, and PPDI molecules in the gas phase. Additionally, for all molecules the N–H and I–H bond lengths are depicted in angstroms.

This section aims to discuss the results for molecules in the gas phase and to obtain insights into the geometric and energetic features of the passivators. We investigated neutral PPD, charged p-phenylenediammonium (PPD^2+^), which was obtained by removing two electrons from PPD, and p-phenylenediammonium iodine (PPDI), all in the gas phase. Figure 2 presents a ball-and-stick representation of the PPD, PPD^2+^, and PPDI molecules in their gas phase, indicating the most relevant bond lengths. For PPD, we found distinct lengths of the N– H bonds, with the longest bond indicating a higher susceptibility to break. Furthermore, the neutral PPD molecule has an ionization potential of 2.23 eV and the HOMO–LUMO energy difference (Δ^*HL*^) of 0.53 eV, suggesting its cationic behavior and tendency to donate electrons when compared to typical semiconductor surfaces.

To construct the PPD^2+^ molecule, two electrons were removed from the neutral PPD molecule, motivated by its bicationic feature. Monopole and dipole corrections were applied to ensure accuracy in the energy calculations. The optimized PPD^2+^ molecule shows three equivalent N– H bond lengths of 1.04 Å, with an increase in Δ^*HL*^ of 4.89 eV, indicates enhanced stability compared to the neutral PPD. The addition of two I atoms to form PPDI resulted in charge-neutral configurations, the most stable configuration having I atoms on opposite sides of the PPD molecule. This configuration exhibits a Δ^*HL*^ of 3.39 eV and an ionization potential of 5.60 eV, demonstrating better stability than the PPD molecules.

The cohesive energies of these gas-phase molecules are lower than those of the bulk CsPbI_3_ perovskites due to the stronger covalent bonds in the molecules compared to the ionic bonds in CsPbI_3_. This implies that interaction with CsPbI_3_ surfaces would likely distort the bulk structure more than the molecular structure.

Additionally, Bader charge analysis revealed significant charge transfer in the NH_3_ groups, identifying them as the most active sites in the molecules. In PPD^2+^, the equal lengths of the N– H bonds result in a planar structure. For PPDI, the presence of I atoms attracts charge from NH_3_ groups, stabilizing the molecule. Therefore, horizontal adsorption of PPD molecules on the CsPbI_3_ surfaces is expected to enhance interactions due to the aromatic ring and NH_3_ groups.

### 3.3 Low Energy PPD/CsPbI_3_ Configurations

**Figure 3:**
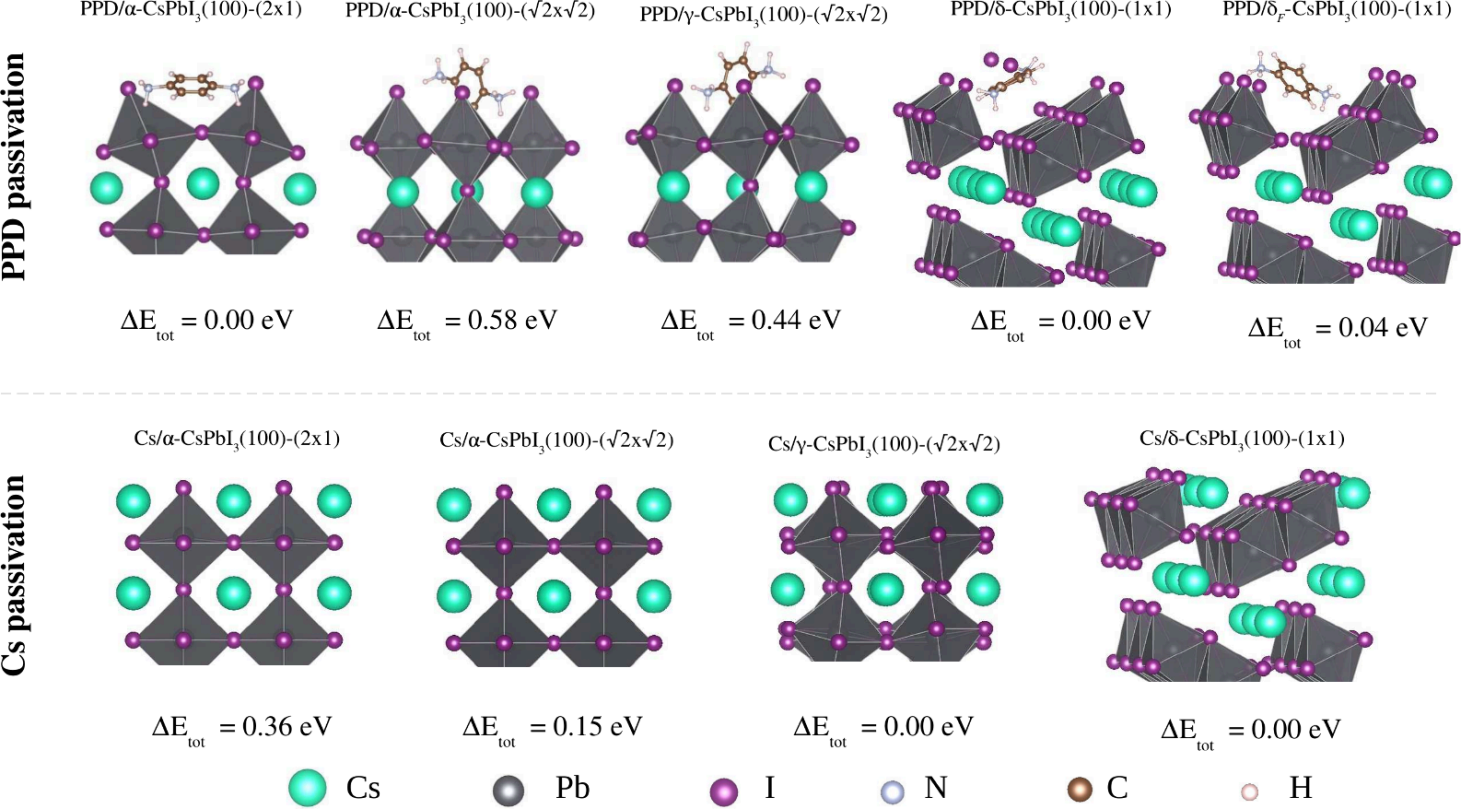
Minimal energy configurations of the CsPbI_3_ surfaces passivated with PPD molecules (top panel) and Cs atoms (bottom panel). The energy difference (Δ*E*) for black phases is with respect to PPD/α-CsPbI_3_(100)-(2 × 1), while the reference of yellow phases is the energy of PPD/δ-CsPbI_3_(100)-(1 × 1).

This section presents an energy analysis to investigate the stability of PPD passivated CsPbI_3_ surfaces. For all initial configurations described in Section 2.2.3, we performed a force optimization process to determine the lowest energy configuration for each surface phase. We rearranged the configurations from lower to higher energy, as shown in Table S14 of the SI file. The minimal energy configurations for PPD/α-(2 × 1), PPD/α-, PPD/γ-, and PPD/δ-(1 × 1) were r5, h4, h2 and r2, respectively, which are depicted in Figure 3.

The low energy configuration of PPD/δ-(1 × 1) shows a fragmentation of its most superficial octahedra characterized by the loss of iodine atoms (there are free I atoms). Thus, we considered an additional optimization protocol for the yellow surface indicated as PPD/δ_*F*_-CsPbI_3_(100)-(1 × 1) (abbreviated as PPD/δ_*F*_-(1 × 1)), where the subscript *F* stands for ”frozen”. This protocol considers a different optimization procedure: (*i*) We first freeze all atoms of the CsPbI_3_ slab while optimizing only the atomic positions of the PPD molecules; (*ii*) Once this relaxation process is reached, it is used as a starting point for a new geometry optimization with the old protocol, that is, optimizing the geometry while freezing only the central part of the slab. For this frozen phase, the relaxed minimal energy configuration corresponds to the configuration *r*3.

The lowest energy configuration among all PPD-passivated black surfaces corresponds to the minimal energy configuration of the relaxed PPD/α-(2 × 1) surface. This configuration has the PPD molecule in an almost horizontal position relative to the slab. By comparison, we point out that the relative energy (*δE*_*tot*_) of the minimal energy configuration of PPD/α- and PPD/γ- surfaces is 0.58 and 0.44 eV, respectively. In both cases, the PPD molecules are in a perpendicular position relative to the surface. Thus, horizontal adsorption acts as a stabilization factor.

The energetics of the PPD-passivated yellow surfaces (PPD/δ-(1 × 1) and PPD/δ_*F*_-(1 × 1)) are analyzed separately from the black phases due to their different stoichiometries. The optimized structures resulting from the frozen and nonfrozen protocols for the yellow phase have nearly degenerate lowest energy configurations, with a slight gain of 0.04 eV for the frozen protocol over the nonfrozen protocol. In the case of PPD/δ-(1 × 1), the lowest energy configuration shows the PPD molecule positioned parallel to the inclined face of the chain of surface bioctahedrons forming the surface. As mentioned above, two free I atoms surround the PPD molecule, which were extracted from the top of the chain of bioctahedrons.

In the relaxed PPD/δ_*F*_-(1 × 1) case, the position of the PPD molecule is perpendicular to the chain of distorted bioctahedrons. One NH_3_ group is near the I atoms of the central part of the distorted chain of bioctahedrons, while the other NH_3_ group is close to the I atom located at the top of the next chain of distorted bioctahedrons. These results are not surprising, as these configurations favor interaction between the *sp*^3^ orbitals of the NH_3_ groups and the *p* orbitals of I on the CsPbI_3_ surface. Therefore, since the frozen configuration preserves all surface iodine atoms, unlike the other phase, this lower energy difference can be interpreted as the energy required to remove an iodine surface atom in the presence of PPD passivators in the yellow phase. In other words, PPD surface molecules degrade the yellow phase due to the easy removal of I surface atoms.

Assessing the distortions generated between PbI_6_ octahedra is an effective way to analyze the consequences of the passivation process. We performed such analysis by measuring the average angle between consecutive octahedra, denoted θ_av_^PbIPb^. The black phases PPD/α-(2 × 1) and PPD/α- have values of 169.26° and 166.42°, respectively. On the other hand, for the Cs passivation of the same phases, θ_av_^PbIPb^ takes the values of 176.15° and 176.74°, respectively. For the γ phases, we observe a value of θ_av_^PbIPb^ of 159.57° for PPD/γ-, while for Cs/γ- we have 163.06°. For the yellow phase Cs/δ-(1 × 1), the value of θ_av_^PbIPb^ is 92.38°, however for PPD/δ-(1 × 1) and PPD/δ_*F*_-(1 × 1) we obtained 93.07 and 94.01° respectively. Thus, in all cases, PPD passivation distorts octahedra more than Cs passivation. In the case of PPD passivation, the angular distortion increases as the octahedron layers approach the surface as a natural response to the rearrangement of PPD molecules on the surface. On the other hand, yellow-passivated surfaces are much less susceptible to octahedral distortions than black surfaces. We attribute this behavior to the connections between the PbI_6_ octahedra, which are face-shared in the yellow phase, whereas the black phases have only corner-shared octahedra.^55^

### 3.4 Surface Energy Formation Analysis

The different stoichiometries of the black and yellow models prevent a direct comparison of their relative energies to explore the most stable passivated phase. Thus, it is suitable to examine the surface formation energy (*E*_*F*_^*S*^) to assess the relative stability between phases, even for phases with different stoichiometries and passivators.

The descriptor *E*_*F*_^*S*^ is calculated using the following equation:^56^
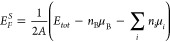
3where *A* represents the surface area, *E*_*tot*_ denotes the total energy, *n*_*B*_ indicates the number of bulk unit cells (i.e., the number of CsPbI_3_ formula units), μ_*B*_ stands for the bulk chemical potential calculated as the total energy per unit cell of the bulk structure, *n*_*i*_ represents the number of binary constituents bearing to the surface, and μ_*i*_ refers to their respective chemical potentials, where *i* = CsI, PbI_2_, or PPDI. The bulk chemical potential phase (α, γ, or δ) should match that of the inner region of the investigated system. The binary surface constituents are CsI, PbI_2_, and PPDI. For the first two, we consider the lowest energy bulk phases,^65^ namely a salt structure for CsI and a van der Waals crystal structure for PbI_2_, representing CsI- and PbI_2_-rich conditions. However, the chemical potential of PPDI was determined from the lowest-energy molecules in the gas phase for simplicity, as the original experiment considers PPDI in isopropyl alcohol solution.^34^

For clarity, we illustrate in the following the evaluation of *E*_*F*_^*S*^ in some systems. For the case of PPD molecules passivating the PPD/α-(2 × 1) surface, the formula unit for this system is Cs_8_Pb_10_I_30_(PPD)_2_. Here, we consider as binary constituents two PPDI molecules and two PbI_2_ formula units on the surface (one of each for both sides of the slab). There are eight unitary bulk formulas and it is important to use the corresponding bulk chemical potential, in this case the α one. Thus, after applying the eq 3, we obtain the value of 24.91 meV/Å^2^. Additionally, the same formula unit and binary constituents occurs for PPD molecules saturating the γ-phase, as for the α phase. However, it is only important to ensure the use of the corresponding bulk chemical potential for the γ phase. Furthermore, the yellow phase has a different formula unit (Cs_8_Pb_12_I_36_(PPD)_2_); thus, the surface binary constituents are two PPDI molecules, four PbI_2_ beyond the existence of eight bulk formula units. In the cases of Cs passivated surfaces, each PPDI binary constituent is replaced by two CsI units.

**Figure 4:**
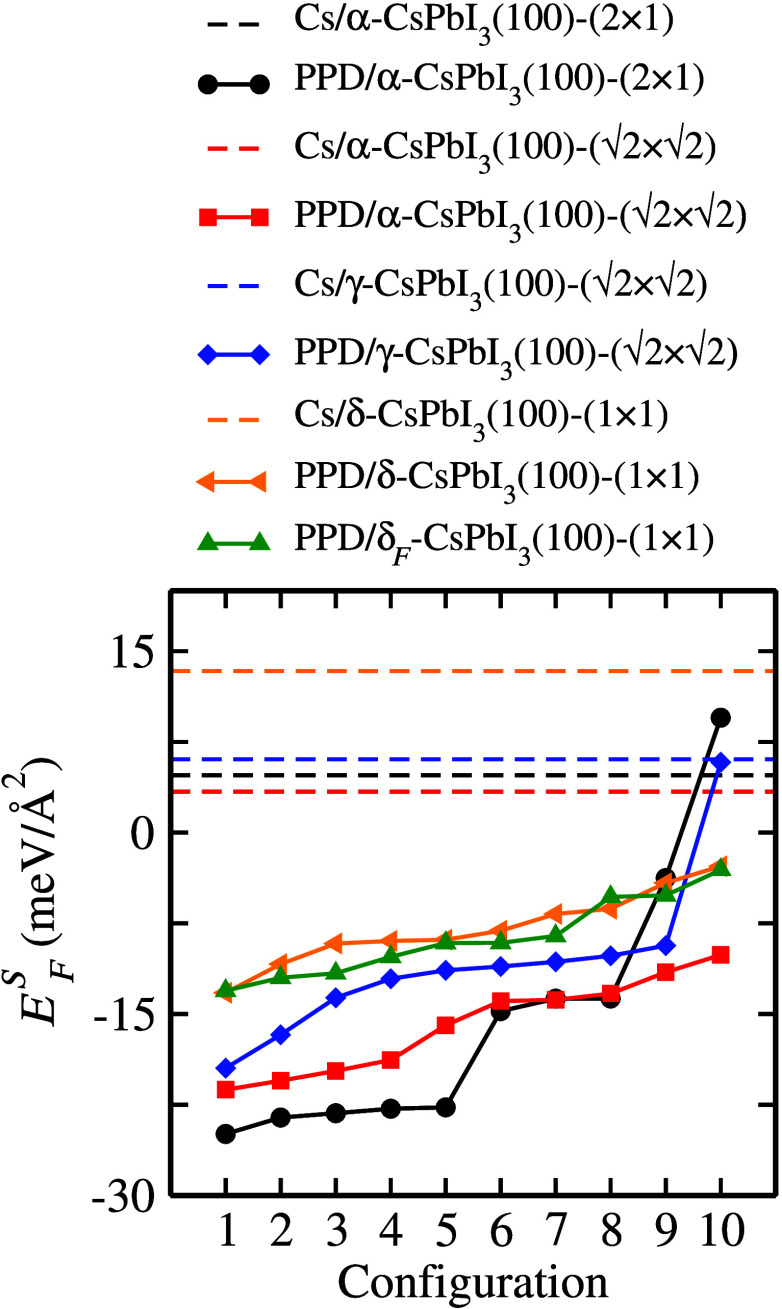
Surface formation energy of the passivated CsPbI_3_ surfaces with the PPD molecules or Cs species.

Figure 4 shows *E*_*F*_^*S*^ for each optimized configuration of the black and yellow passivated surfaces. These systems are sorted from lowest to highest Δ*E*_*tot*_, that is, the configurations 1 correspond to the minimal energy configurations shown in the upper panels of Figure 3. Furthermore, the *E*_*F*_^*S*^ for the Cs passivated surfaces appears as dashed lines. The lowest value of *E*_*F*_^*S*^ among all analyzed surfaces occurs for the lowest energy configuration of PPD/α-(2 × 1), although in its bulk configuration the α-CsPbI_3_ phase has a higher *E*_*F*_ compared to the bulk phases γ- and δ-phases, as we can see in the Δ*E*_*F*_ column of Table 1. Thus, PPD passivation exchanges the relative stability among the phases. This result corroborates the experiments by Ding et al.,^34^ that indicate that the PPDI molecules increase the stability of the α-phase, which opens the possibility of future photovoltaic applications.

Moreover, all Cs passivated surfaces have positive *E*_*F*_^*S*^, indicating instabilities as there is an energetic preference for the system to dissociate into bulks and binary constituents. It is important to stress that the surface saturation with Cs atoms is expected to be the natural saturation once Cs appears in the composition of CsPbI_3_ systems. We obtained positive values of *E*_*F*_^*S*^ independently of the phase of the surfaces. This result indicates the instability of this surface and the necessity of searching for different surface passivators.

**Figure 5:**
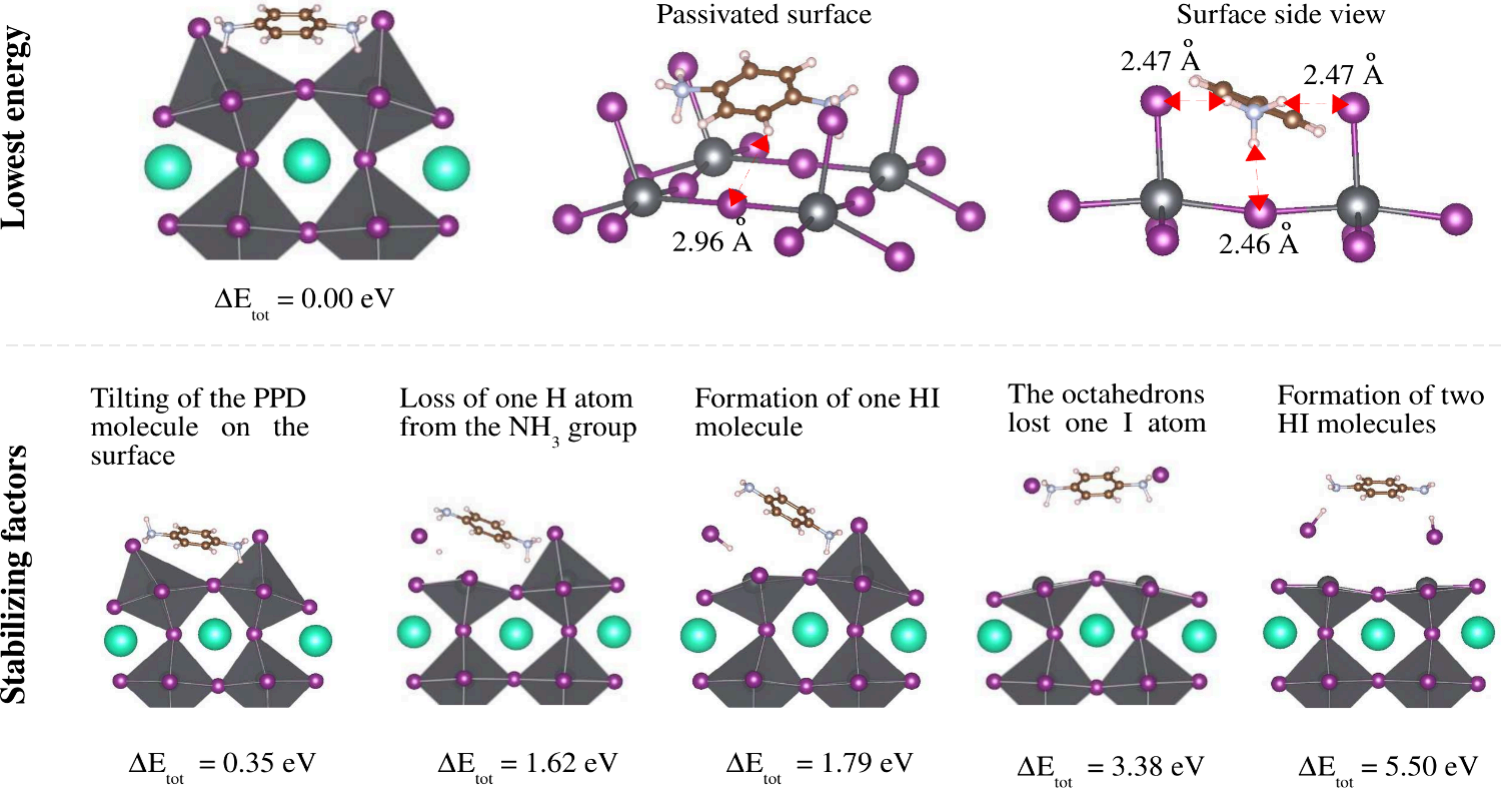
Surface morphology of the minimal energy configuration of the PPD/α-CsPbI_3_(100)-(2 × 1) surface (top panel). Stabilizing factors of higher energy configurations in the PPD passivation process of CsPbI_3_ surfaces (bottom panel).

In general, flat and sharp increases in *E*_*F*_^*S*^ correlate well with changes in the relative total energy. For example, in the case of PPD/α-(2 × 1), the notable changes in *E*_*F*_^*S*^ for configurations 5, 6, 8, 9, and 10 are consequences of the changes in the atomic configuration revealed by stabilizing factors. These factors are shown sequentially from left to right in the bottom panel of Figure 5 and will be discussed in detail in the next section.

### 3.5 Surface Morphology of the PPD Passivation in Black Phases

The adsorption of PPD molecules induces significant geometrical distortions near the surfaces. The reason for the high distortions in the perovskite region is the large hardness of the chemical bonds mentioned above in the PPD molecule due to its dominant covalent character, compared to the dominant ionic bonds in bulk perovskite CsPbI_3_. In addition, there are other interaction mechanisms at work. For example, the two NH_3_ groups of PPD molecules are active for charge transfers, effectively acting as monovalent cations similar to Cs atoms. They are responsible for the interaction with the surface through Coulomb interactions from their charge transfers, in addition to forming hydrogen bonds with surface atoms I. Moreover, the central aromatic ring also binds to the surface through van der Waals interactions. Thus, there are numerous mechanisms of interaction between the surface and the PPD molecule.

As mentioned previously, the lowest energy configuration of the PPD-passivated black surfaces corresponds to the minimal energy configuration of the relaxed PPD/α-(2 × 1). In this configuration, the aromatic ring of the PPD molecule is almost parallel to the surface. Each PPD molecule is located in the center site left by four PbI_6_ octahedra, where their top I atoms form a square and are the same height as the N atoms of the PPD molecule, as shown on the central side of the top panel of Figure 5. These octahedra are oriented outward, ensuring an excellent arrangement of the PPD molecule, where each N– H bond of the two NH_3_ groups points directly to its nearest I atom. Furthermore, the average distance between H and I atoms is 2.47 Å, as shown on the right side of the top panel of Figure 5.

In the lower panel of Figure 5, we present some configurations of PPD/α-(2 × 1) with higher energy. These configurations reveal stabilizing factors of the PPD passivating process. The tilt of PPD molecule may result in the NH_3_ atoms not pointing at the I atoms, leading to an energy gain of 0.35 eV. Additionally, it may occur that the orientations of PPD molecule lead to one of its NH_3_ groups being close to the I atoms, causing an H atom to be extracted from the PPD molecule, resulting in an energy gain of 1.62 eV, or a free HI molecule could be formed, leading to an energy gain of 1.79 eV.

On the other hand, we found other configurations with high energies falling into local minima of the potential energy surface, thus meeting the criteria of zero forces. However, these configurations could exhibit atypical physicochemical properties. For example, our simulations revealed a configuration in which the PbI_6_ octahedra lose superficial I atoms and the PPD molecule attracts them. This process leads to an unstable surface with an increase in the total energy of 3.38 eV, as can be seen in Figure 5. Alternatively, it could happen that PPD molecule loses an H atom from its two NH_3_ groups, forming two free HI molecules, resulting in an energy increase of 5.50 eV. These last two scenarios are practically unstable but provide some insight into what can occur in the PPD passivation process; however, the relatively high energies (>3 eV) indicate processes triggered only at high temperatures.

In contrast, the yellow phase has the lowest energy configuration with decoupled iodine atoms from the surface. In fact, the two lowest-energy configurations of the frozen and nonfrozen optimization protocols are almost degenerate in energy (see Figure 3). Thus, PPD molecules cause instabilities in the yellow phase but not in the black phases.

### 3.6 Mechanism of the Interactions between the Passivator and Surface

To elucidate the enhanced stability of surface passivation of the black phase PPD/α-(2 × 1) relative to alternative configurations, we conduct a Bader effective charge analysis.^66^Table 3 presents the average Bader effective charge (Q̅) per atomic species and passivators for our set of passivated surfaces with the lowest energy. Here we compare the two types of passivation, that is, by Cs atoms or PPD molecules, to extract some important clues about the electronic charge distribution and possible bonds between the components and the surface. In all cases, there are no significant changes in the positive effective charge in Cs and Pb atoms of the slab between the two passivation cases, showing absolute value differences of less than 0.01 *e*, except for an increase of 0.03 *e* on the Pb atoms in PPD/γ- compared to the Cs passivation case.

**Table 3:** Effective Bader Charges of the Passivated Surfaces of CsPbI_3_^a^

surface	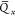	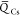	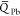	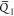
Cs/α-CsPbI_3_(100)-(2 × 1)	0.87	0.87	0.91	–0.61
PPD/α-CsPbI_3_(100)-(2 × 1)	0.67	0.86	0.92	–0.59
Cs/α-CsPbI_3_(100)-	0.87	0.87	0.90	–0.61
PPD/α-CsPbI_3_(100)-	0.68	0.86	0.93	–0.59
Cs/γ-CsPbI_3_(100)-	0.85	0.85	0.92	–0.61
PPD/γ-CsPbI_3_(100)-	0.68	0.85	0.93	–0.59
Cs/δ-CsPbI_3_(100)-(1 × 1)	0.82	0.81	0.94	–0.59
PPD/δ-CsPbI_3_(100)-(1 × 1)	0.70	0.82	0.94	–0.57
PPD/δ_*F*_-CsPbI_3_(100)-(1 × 1)	0.70	0.82	0.94	–0.57

a Averages of effective Bader charges on the passivator (, where *x* = Cs surface atoms or PPD molecules), on Cs atoms inside the bulk , and on Pb  and I atoms . Here, we show half of the effective charge of the PPD molecules. All values are in units of fundamental electronic charge *e*.

The most remarkable difference between the two types of passivation is the positive effective charge on each passivator. Since the PPD cation has an oxidation number of 2^+^, to compare its effective charge with that of the Cs cation, we report half the effective charge on the PPD cations. In all cases, we observed a higher concentration of positive charge on the Cs superficial atoms compared to the PPD cations. The main differences occur in the black phase α-(2 × 1) with an increase of 0.20 *e*. On the other hand, we have a smaller difference of 0.11 *e* on the δ-(1 × 1) passivated surface. Additionally, in all cases in the Cs passivation case, there is a slight excess of effective negative charge on the I atoms, ranging from −0.01 to −0.02 *e*, compared to their corresponding PPD passivation case. Thus, having a more effective charge on the Cs surface atoms and the I surface atoms of the octahedra indicates a greater tendency to have ionic bonds between them, as in the case of Cs passivation. On the other hand, for the case of PPD passivation, a lower effective charge on the surface atoms of PPD molecules and I indicates a lesser contribution of ionic bonding, which leads to the enhancement of other types of bonds, such as covalent or van der Waals bonds. Therefore, our results suggest that the presence of PPD molecules produces a mixture of atomic bond types, enhancing surface stability.

We performed a difference charge density (Δρ) analysis in order to spatially visualize the presence of bonds between passivator and surface I atoms. Δρ is defined by the following equation:
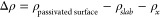
4where, ρ_passivated surface_ is the charge density of the passivated surface, ρ_*slab*_ denotes the charge density of bare slab and ρ_*x*_ is the charge density of the passivators.

**Figure 6:**
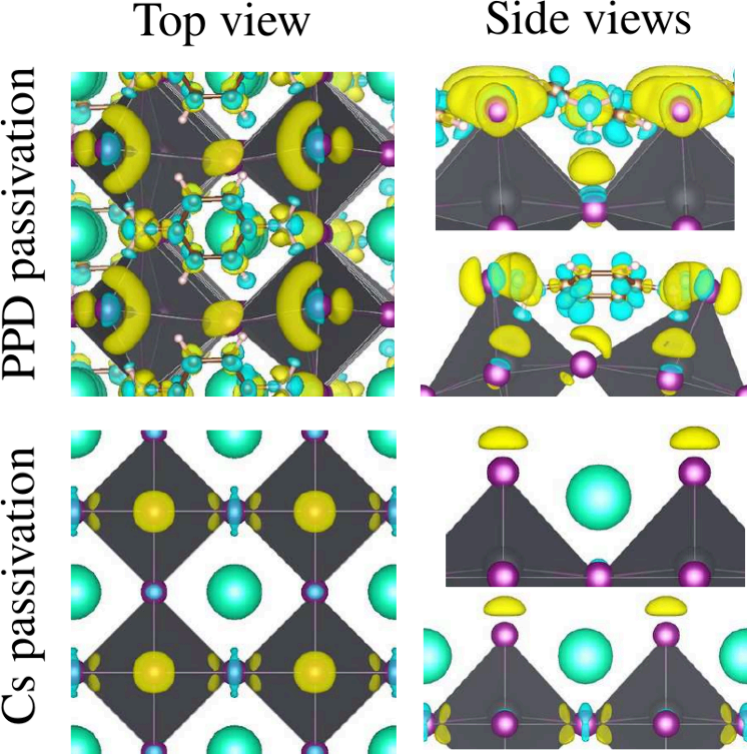
Plot of the difference in the charge density of the *x*/α-CsPbI_3_(100)-(2 × 1) passivized surface, where *x* = PPD or Cs, which denote the two kind of passivations analyzed here. Yellow areas denote charge gain, and blue areas charge loss. The isosurface was taken as 0.002 bohr^–3^.

We examine the Δρ of the lowest energy configuration of the relaxed surfaces PPD and Cs passivated α-(2 × 1) surfaces, which are illustrated on the Figure 6. The yellow zones indicate the gain in charge density, and the blue zones indicate the loss in charge density. For the case of PPD passivation, in the Δρ inside of the PPD molecule, blue, we can see the presence of covalent bonds between the π orbitals on each C atom of the aromatic ring. Furthermore, we can observe the charge density surrounding the H of the two NH_3_ groups, this confirming an orbital hybridization *sp*^3^ on the N atoms. On the other hand, on the surface I atoms we can see the presence of blue areas, also, indicating a density of charge on the *p*-orbitals of those atoms. When the passivated surface is built up, the density of the charge is rearranged in a way that forms a new distribution of the charge, denoted in yellow areas, concentrating the charge on the *p* orbitals of I atoms and on the N atoms of the PPD molecule.

In fact, passivation of the CsPbI_3_ surfaces with organic molecules provides a rich chemical environment, allowing covalent bonds within the molecule and hydrogen bonds between the H atoms of the PPD molecule and the I atoms of the surface. In particular, since PPD is placed horizontally but with a slight inclination, this configuration increases the density of charge of seven hydrogen bonds. Six of them are in the reactive group NH_3_ at the ends of the PPD molecule, and the other is located in the closest part of the aromatic ring closest to the surface, as shown in the top panels of Figure 5. Thus, these increases in charge density lead to an enhancement of the covalent character of those hydrogen bonds, which could be the reason for the improved surface stability. On the other hand, the passivated case Cs is simple. We observe an accumulation of density charge only on the I atoms, which corroborates that the surfaces Cs atoms bond to the surface mainly by ionic bonds.

### 3.7 Electronic Properties

**Figure 7:**
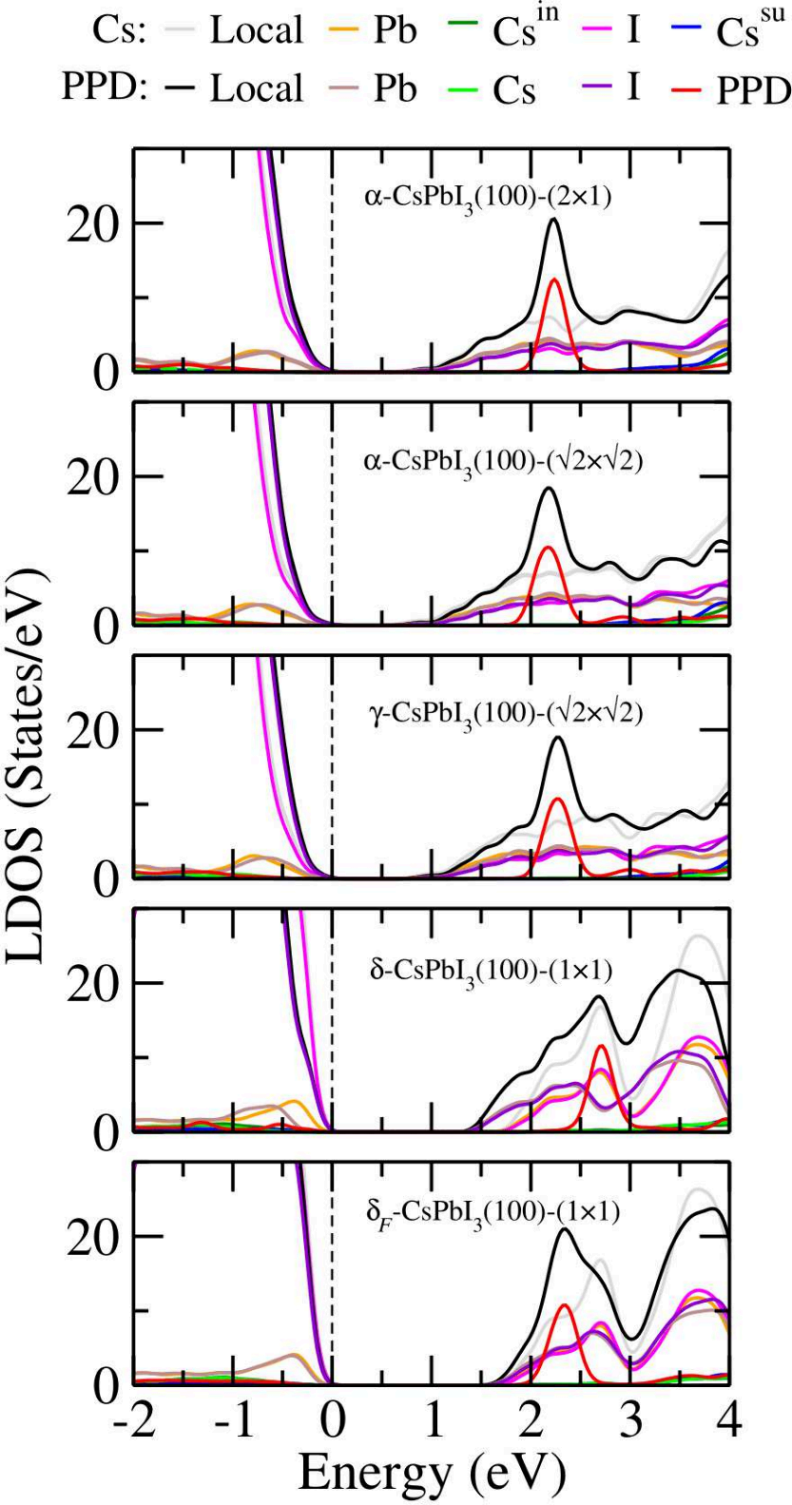
Local density of states (LDOS) and its decomposition per chemical species for the PPD and Cs passivated surfaces. The dashed lines indicate the case of passivated surfaces with Cs. The calculations were performed by using the PBE+D3+SOC method.

In this section, we examine how the electronic characteristics of the most stable configurations are influenced by the PPD passivation, contrasting them with the outcomes of the Cs passivation. Figure 7 illustrates the local density of states (LDOS) and its breakdown based on chemical species and the passivation molecule. LDOS was determined using the PBE+D3+SOC approach.

For all surfaces, the electronic states on the valence band maximum (VBM) are dominated by the presence of the *p*-states of the I atoms. However, for the electronic states on the conduction band minimum (CBM), we observe a maximal contribution of the *s*-states of Pb and *p*-states of I atoms at the same time.^50^ For all black surfaces and the yellow frozen surface, the contribution of the atomic species Pb, Cs, and I to the LDOS is quite similar, with the exception of the contribution of the passivator. For the case of PPD passivation, the electronic states of PPD molecule are located inside of the conduction band, almost 1.5 eV above from the CBM, in the case of black surfaces, while for the yellow frozen one, the same thing happens, but those states are 0.6 eV above from the CBM. On the other hand, for the passivation Cs for all surfaces, *s*-states of the surface Cs atoms are extremely high above the CBM.

**Figure 8:**
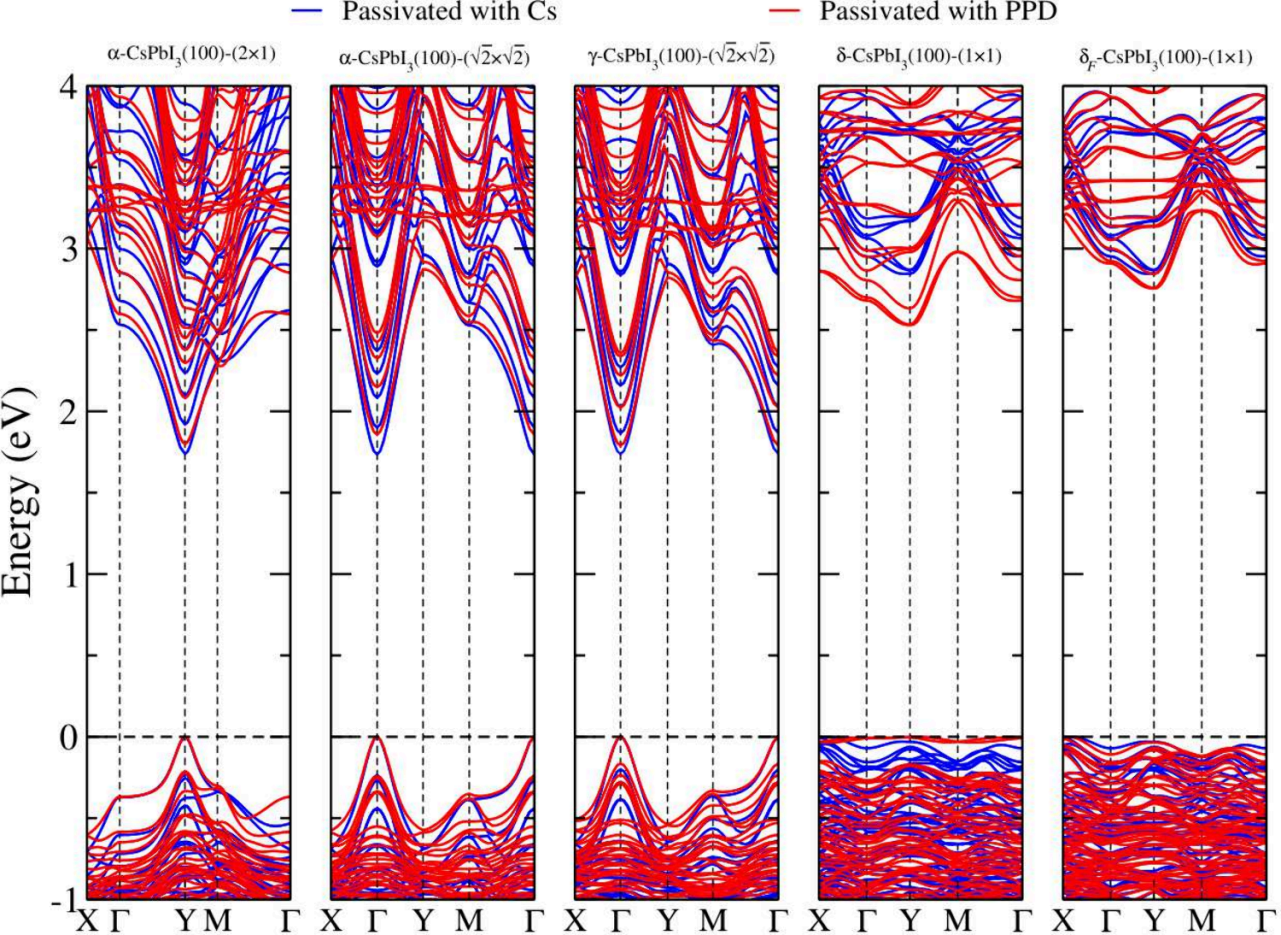
Electronic band structures of the PPD and Cs passivated surfaces. The Fermi level is set to zero. All band structures have been calculated using the PBE+D3+SOC+HSE+QC method.

For the yellow surface, we observe a remarkable difference in LDOS between the two passivation cases, in terms of the size of the band gaps and their shape. This difference is due to fractures in the surface octahedra passivated with PPD, as shown in Figure 3, where some I atoms were removed from the octahedra by the PPD molecule. In contrast, in the Cs passivation, we do not observe any distortion of the octahedrons. Therefore, the use of PPD molecules as a passivator of CsPbI_3_ surfaces does not change the behavior of LDOS near the band gap. In Figure 8, we show the electronic band structure for the same passivated surfaces presented at the beginning of the section. As expected, the bands corresponding purely to the bare slabs in the two kinds of passesivation are almost overlapping each other. With the exception of the yellow surfaces, the bands have a different behavior, as previously discussed.

All passivated surfaces exhibit a direct band gap transition, with the exception of the yellow surfaces Cs/δ-(1 × 1) and PPD/δ_*F*_-(1 × 1) yellow surfaces, which show an indirect band gap from Γ to the Y point. The gaps of α-(2 × 1) and α- describe the same surface, and we observe that the position of the band gap is different. This is due to the folding of the Brillouin zone effect. The first band gap is located at the Y point, while the second band gap is at the Γ-point. Likewise, the band structure of α-, and γ- surfaces is very similar, but the bands of the latter are slightly more elongated and the values of their band gaps are slightly larger than those of the others. The last effect is well-known; its origin is due to the angular distortion between consecutive octahedrons on γ- surfaces, which alters the hybridization between the Pb 6s orbitals and the I 5p orbitals, affecting the band gap value.^67^ Furthermore, for all PPD passivated surfaces, we observe quasi-flat conduction bands that are associated with the localized orbitals of the PPD molecule at higher energy levels. This finding confirms that the PPD molecule does not modify the electronic band structure near the band gap.

The band gap value is a key parameter for photovoltaic applications due to its important role in the absorption of sunlight. As the purpose of this work is to investigate the CsPbI_3_ bulk (*B*) passivation process using of our slabs (*S*) models, there is a inherent change in the band gap values due to the quantum confinement. To calculate an accurate band gap for passivated slabs we include a term of quantum confinement effect (χ^QC^(*S*)) in the band gap equation as
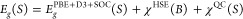
5

For the Cs passivated case, the quantum confinement effect can be calculated directly by computing the band gap of the CsPbI_3_ bulk and subtracting the value from that of the Cs passivated slab (*S*_Cs_) as χ^QC^(Cs)=*E*_*g*_^PBE+D3+SOC^(*B*)-*E*_*g*_^PBE+D3+SOC^(*S*_Cs_). By simple substitution on eq 5, *E*_*g*_(*S*_Cs_)=*E*_*g*_^PBE+D3+SOC^(*B*)+χ^HSE^(*B*)=*E*_*g*_(*B*), leading to a natural normalization respect to the bulk value.

On the other hand, for the PPD passivation case, it is not possible to calculate directly the quantum confinement term (χ^QC^(PPD)), since we do not know the band gap value of CsPbI_3_ bulk passivated with PPD molecules (*E*_*g*_^PBE+D3+SOC^(*B*_PPD_)), because calculating it requires to use an infinite unit cell. However, we perform an estimation of it by calculating the band gaps of some PPD passivated slabs when its thickness increasing and interpolating those values to infinity. In this way, the quantum confinement effect for the PPD passivation case is given by χ^QC^(PPD)=*E*_*g*_^PBE+D3+SOC^(*B*_PPD_)-*E*_*g*_^PBE+D3+SOC^(*S*_PPD_), where *E*_*g*_^PBE+D3+SOC^(*S*_PPD_) represents the band gaps values of PPD passivated slabs. As expected, the values of χ^QC^(Cs) and χ^QC^(PPD) are equal, then the quantum confinement value is independent of the of passivator, for more details see the SI file. By substitution on eq 5, the band gaps of PPD passivates slabs can be calculated as
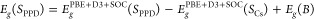
6which corresponds a difference of the band gaps between the two kinds of passivations but normalizing respect to the bulk, allowing us to properly analyze the effect of the PPD passivation on the band gap values.

In Table 4 shows the band gap values of our set of lowest energy passivated surfaces obtained by PBE+D3, PBE+D3+SOC, and scissor operator methods that include the hibryd HSE for the bulk case and quantum confinement rigid shifts. For perovskite materials, it is desirable to have a band gap in the range of 1.1 to 1.8 eV for photovoltaic applications.^30^ However, the final calculated band gap (*E*_*g*_) using the scissor operator method shows that the set of black passivated surfaces has band gap values ranging from 1.73 to 1.86 eV. We observe that the differences between the band gaps for the same passivated black surface but with different passivators are 0.07, 0.13, and 0.05 eV for the surfaces α-(2 × 1), α-, and γ- surfaces, respectively. Thus, the use of PPD as a passivator does not significantly modify the band gap values of CsPbI_3_ with black phases.

**Table 4:** Calculated Electronic Band Gaps of Passivated Surfaces of CsPbI_3_^a^

phase	*E*_*g*_^PBE+D3^	*E*_*g*_^PBE+D3+SOC^	χ^SOC^	χ^HSE^(*B*)	χ^QC^	*E*_*g*_
Cs/α-CsPbI_3_(100)-(2 × 1)	1.49	0.64	–0.85	1.36	–0.27	1.73
PPD/α-CsPbI_3_(100)-(2 × 1)	1.55	0.71	–0.84	1.36	–0.27	1.80
Cs/α-CsPbI_3_(100)-	1.47	0.63	–0.84	1.36	–0.26	1.73
PPD/α-CsPbI_3_(100)-	1.66	0.76	–0.90	1.36	–0.26	1.86
Cs/γ-CsPbI_3_(100)-	1.63	0.83	–0.80	1.09	–0.18	1.74
PPD/γ-CsPbI_3_(100)-	1.74	0.88	–0.86	1.09	–0.18	1.79
Cs/δ-CsPbI_3_(100)-(1 × 1)	2.55	2.05	–0.50	0.76	0.03	2.85
PPD/δ-CsPbI_3_(100)-(1 × 1)	2.27	1.74	–0.53	0.76	0.03	2.53
PPD/δ_*F*_-CsPbI_3_(100)-(1 × 1)	2.47	1.96	–0.51	0.76	0.03	2.75

a The band gap (*E*_g_) was estimated as *E*_g_ = *E*_g_^PBE+D3^ + χ^SOC^ + χ^HSE^(*B*) + χ^QC^ (or equivalent as in eq 5 or 6), where *E*_g_^PBE+D3^ is the band gap calculated by using the PBE+D3 method, χ^SOC^ is the band gap decreasing from *E*_g_^PBE+D3^ employing the spin-orbit coupling (SOC), the values of χ^HSE^(*B*) were taken from the bulk calculations reported on Table 2, and χ^QC^ is the change of the band gap due to the quantum confinement. All values are presented in electronvolts.

### 3.8 Absorption Coefficient

**Figure 9:**
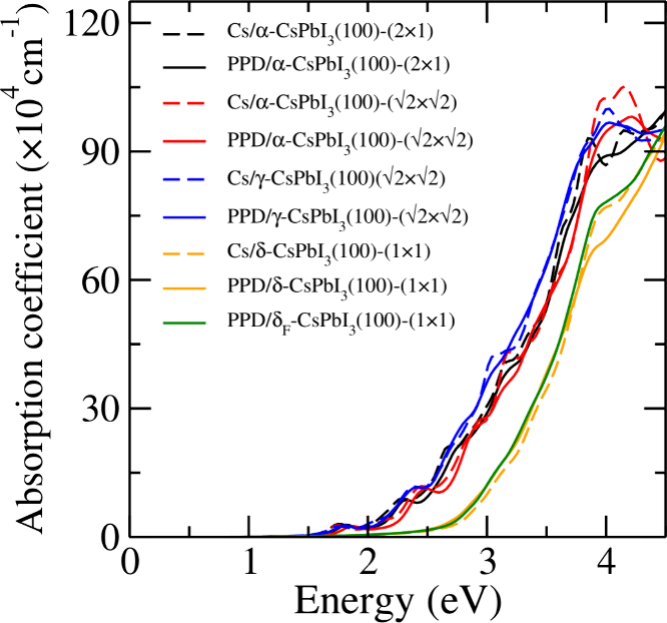
Absorption coefficient for the passivated surfaces. The values were calculated using the PBE+D3+SOC+HSE+QC method and shifted on the energy axis by using the scissor operator method described in Table 4.

In this section, we discuss the absorption coefficient of the PPD and Cs passivated surfaces analyzed in the previous section. The values of their absorption coefficient are calculated using the PBE+D3+SOC+HSE+QC method and are shown in Figure 9. For each system, we can see that the shapes of the curves of the two kinds of passivations overlap each other almost. This is due to the electronic states of the PPD molecule being above the CBM. Thus, the low-energy optical transitions correspond purely to the transitions between the electronic states of the bare slabs. For black passivated surfaces, the absorption coefficient begins with energies close to 1.73 eV, which corresponds to the values of the band gaps reported on Table 4. Thus, the PPD molecule becomes optoelectronic transparent, which is desirable for the good performance of CsPbI_3_ perovskites as photovoltaic materials.

## 4 Insights into the PPD Passivation

Outstanding research in perovskites has demonstrated the potential of surface passivation in improving the operational efficiency of solar energy devices.^35^ In this context, Ding et al.^34^ demonstrated the use of the PPD molecule as a surface passivator for CsPbI_3_ perovskites, preventing the transition from black to yellow phase due to moisture infiltration. However, a deep atomistic description of the reasons behind the successful surface passivation still presents a substantial knowledge gap and will be helpful for the surface passivation process as a whole.

First, our first-principles calculations corroborate the findings of Ding and co-workers, showing that PPD passivation stabilizes the black α-CsPbI_3_ perovskite against the yellow phase. As our results are aligned with experimental measures, we believe that the model we use captures the most relevant features for stability purposes. However, the atomistic description of the surface passivation mechanism is the main aid of our work, which goes deeper than experimental analysis. Generally, our simulations reveal a high influence of the passivator geometries on the stability of perovskites.

Specifically, we show that the PPD-passivated α phase is more stable than the other black phase (γ) and than the yellow (δ) phase. Thus, PPD passivation completely reverses the total energy trends of the bulk phases. A detailed atomistic analysis shows that, depending on the orientation of the PPD molecule on the surface, some surface I atoms can be released from the octahedra due to interactions with the NH_3_ group, leading to structural defects that could compromise the stability and photovoltaic performance of perovskites. This specific instability is much more prominent in the yellow phase and occurs in the black phase at high energies (and thus temperatures). Moreover, the electronic states PPD demonstrate transparency around the band edge region of the electronic band structure of CsPbI_3_ perovskites. In other words, the electronic states derived from PPD are far in energy from both the valence band maximum and the conduction band minimum, thus preserving the inherent absorption properties of the pure CsPbI_3_ bulk material.

In the PPD-passivated α phase case, the interaction between the PPD molecule and the perovskite surface occurs through the reactive groups NH_3_ and surface I atoms. Based on our results, we believe this interaction is primarily due to iodine–hydrogen bonding (NH ···I) for the following reasons: (i) The average distance between the nearest I atoms and H is 2.47 Å, matching the reported distance for iodine–hydrogen bonding in perovskites.^68^ (ii) Our charge density difference analysis reveals that the charge distribution between the NH_3_ group of the PPD molecule and the most superficial I atoms corresponds to that of iodine–hydrogen bonding. (iii) The effective charge analysis shows that, in the case of PPD passivation, the average effective charge on I and PPD decreases compared to that in the Cs passivated case, indicating a nonionic bonding nature. This confirms an enhancement of the covalent nature of the iodine–hydrogen bonding between PPD molecules and the perovskite surface.

These characteristics make the PPD molecule a highly effective passivation agent for α-CsPbI_3_ perovskites. Our detailed atomistic analysis reveals the fundamental mechanisms that ensure energetic stability while maintaining the outstanding optoelectronic properties of CsPbI_3_ in its black phase.

## 5 Conclusion

In this work, we report a theoretical study based on DFT calculations to investigate the PPD passivation process of α-, γ- and δ-CsPbI_3_ perovskites. To achieve this, we first investigated the CsPbI_3_ perovskites in their bulk phases. Since several experimental works report different structural parameters of these structures in the literature, we calculated the average values of the lattice constants and compared them with those of our optimized structures. We obtained relative percent errors of less than 0.1%, 2.12%, and 0.57% for the α-, γ-, and δ-phases, respectively. Furthermore, based on the analysis of the cohesive and formation energy, we found that the most stable phases are, in sequence, the δ, γ, and α phases, which is consistent with the order of phase transitions reported for CsPbI_3_ perovskites.

In addition, we precisely replicated the average band gap values obtained from the experimental measurements. This was achieved by initially computing the band gap values using the PBE+D3 and PBE+D3+SOC methods. Then, we calculated the increment in the band gaps caused by considering hybrid functional HSE calculations, where we linearly interpolated the fraction of Hartree–Fock exchange until reaching the desired experimental band gap values. Finally, using the scissor operator method, we exactly replicate the average values of the band gaps for each bulk phase of CsPbI_3_ perovskites.

On the other hand, we performed an analysis of the PPD and PPDI molecules in their gas phases. Based on the ionization potential and the values of the HOMO–LUMO energy difference, which are higher for the PPDI case, we found that the PPDI molecules are more stable than the PPD ones. This indicates a greater tendency for the PPD molecule to bind to I atoms, as occurs in the passivation process. Furthermore, since the cohesive energy of the PPD molecules is stronger than that of the bulk perovskites CsPbI_3_, the latter undergoes major distortions in the passivation process.

For each bulk phase, we built slab models with a width of a few octahedral layers, with (100) surfaces and CsI terminations, extracted from our bulk optimized structures. By means of surface energy analyses, we demonstrated that the α-CsPbI_3_ phase is more stable than the γ-CsPbI_3_ and δ-CsPbI_3_ phases when their surfaces are passivated with PPD molecules, that is, passivation with PPD molecules reverts the energetic preference between the α-, γ- and δ-phases.

Furthermore, the orientation of the PPD molecule plays a crucial role in surface passivation. Based on effective charge and difference density charge analyses, our results show that when the aromatic ring of the PPD molecule is almost parallel to the surface, the interactions between PPD molecules and PbI_6_ surface octahedra are enhanced, which strengthens the covalent character of hydrogen bonds between NH_3_ groups and I atoms. This generates surface reinforcement, and we believe that this is the reason why PPD passivation could prevent moisture and heat-induced structural stress, thus avoiding the degradation of the black phases to the yellow phases. On the other hand, we found that electronic states of PPD molecules have very high energies, almost 1.5 eV higher than the minimum of band conduction for black phases, whose bands are similar to flat ones. This leaves the band gap region unchanged. Furthermore, the absorption coefficient for all phases CsPbI_3_ remains almost unchanged.

Our DFT simulations confirm the efficacy of PPD passivation in stabilizing black α-CsPbI_3_ perovskites against transitions to the yellow phase, consistent with experimental observations. The unique covalent properties of the PPD molecules, coupled with their surface orientation, reduce structural defects and enhance hydrogen bonds. This contributes significantly to improved stability and optoelectronic characteristics, which are critical for efficient solar energy applications. These findings not only validate the effectiveness of PPD as a passivator, but also open avenues for exploring other passivators and perovskite materials.

## Supporting Information Available

Additional computational details, computational convergence tests, data employed for the figures, and complementary analyses (PDF)
