# Supplementary material for: Elucidating Black α-CsPbI3 Perovskite Stabilization via PPD Bication-Conjugated Molecule Surface Passivation: Ab Initio Simulations
Source: ACS Appl Mater Interfaces. 2024 Jul 18;16(30):39251–65. doi: 10.1021/acsami.4c05092 (PMC11299153; doi:10.1021/acsami.4c05092)
Supplement: Supplementary file 1 — am4c05092_si_001.pdf [file am4c05092_si_001.pdf]

Supporting Information: Elucidating Black α-$CsPbI_3$ Perovskite Stabilization via PPD Bication-Conjugated Molecules Surface Passivation: Ab initio Simulations.

José E. González,[†] João G. Danelon,[‡] Juarez L. F. Da Silva,[†] and Matheus P. Lima[*,‡]

†*São Carlos Institute of Chemistry, University of São Paulo, P.O. Box* 780, 13560-970, *São Carlos, SP, Brazil*

‡*Department of Physics, Federal University of São Carlos,* 13565-905, *São Carlos, SP, Brazil*

E-mail: mplima@df.ufscar.br

Contents

1 Selected PAW Projectors **S-2**

2 Bulk Parameters **S-2**

3 Molecules in Gas Phase **S-8**

4 Passivated Surfaces **S-12**

4.1 Total and relative energies S-12

4.2 Surfaces formation energies S-16

4.3 Local parameters S-17

4.4 Electronic density of states S-18

4.5 Electronic band structure S-20

4.6 Quantum Confinement S-21

4.7 Absorption coefficients S-23

1 Selected PAW Projectors

Table S1: Computational details of the selected PAW-PBE projectors: number of valence electrons (Z_{val}), electronic configuration of valence states (Valence), and maximum recommended cutoff energy (ENMAX).

Element	PAW-PBE	Z_{val}	Valence	ENMAX (eV)
H	H_GW 21Apr2008	1	$1s^1$	300.00
C	C_GW 28Sep2005	4	$2s^2 2p^6$	413.99
N	N_GW_new 19Mar2005	5	$2s^2 2p^3$	420.90
I	I_GW 12Mar2012	7	$5s^2 5p^5$	175.64
Cs	Cs_sv_GW 23Mar2010	9	$5s^2 5p^6 6s^1$	198.10
Pb	Pb_sv_GW 04Apr2014	22	$5s^2 6s^2 5p^6 6p^2 5d^{10}$	317.19

2 Bulk Parameters

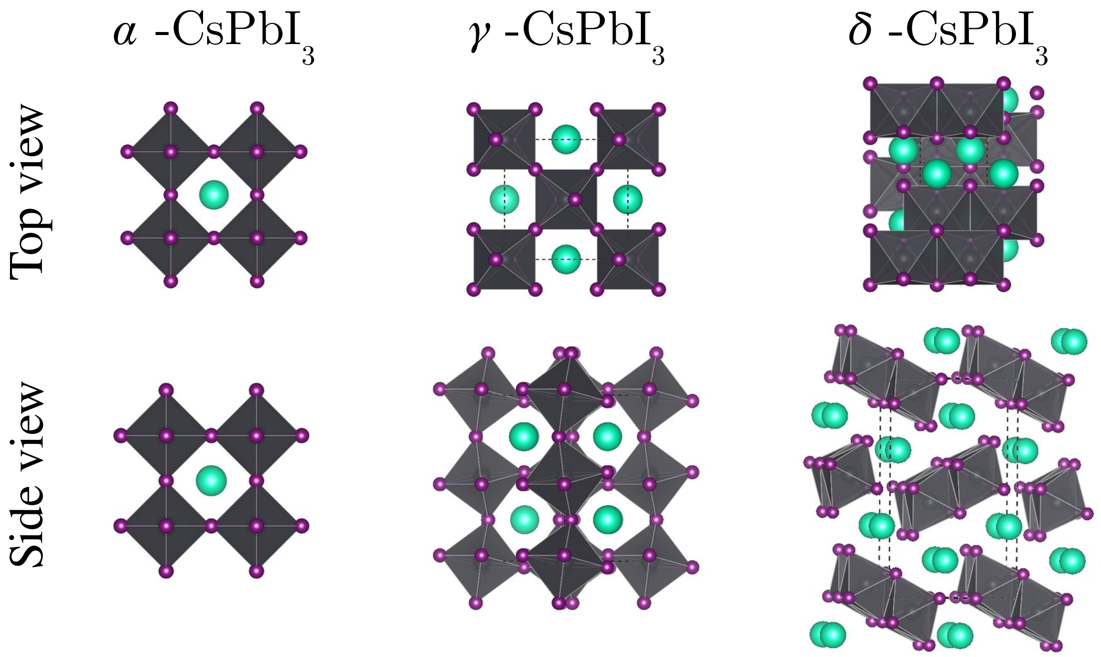

Figure S1: Schematic representation of the structures of α-CsPbI$_3$-$(1\times1\times1)$, γ-CsPbI$_3$-$(\sqrt{2}\times\sqrt{2}\times2)$, and δ-CsPbI$_3$-$(1\times1\times1)$ bulk perovskites. In each case the unit cell is denoted in black dashed lines.

Table S2: Calculated crystallographic parameters of $CsPbI_3$ bulk perovskites on its α, γ and δ, modeling with different supercells. Lattices constants (a_0, b_0 and c_0), angles (α_0, β_0 and γ_0), volume divided by the number of fundamental units ($V_0/f.u.$) and minimal energy divided by the number of fundamental units ($E_{tot}/f.u.$). The unit cell of δ phase cannot be measured in terms of unitary cell of α-cubic phase ($1 \times 1 \times 1$), however since δ phase has the same stecheometry as other phases, we have divided its volume and total energy by 4.

Phase	Supercell	a_0 (Å)	b_0 (Å)	c_0 (Å)	α_0(°)	β_0(°)	γ_0(°)	$V_0/f.u.$ (Å^3)	$E_{tot}/f.u.$ (eV)
α-cub	$1\times1\times1$	6.32	6.32	6.32	90.00	90.00	90.00	252.44	−16.939207
α-cub	$2\times2\times2$	12.64	12.64	12.64	90.00	90.00	90.00	252.17	−16.939468
α-orth	$2\times2\times2$	12.33	12.43	12.79	90.00	90.00	90.00	245.02	−16.990359
α-tric	$2\times2\times2$	12.40	12.42	12.71	90.01	90.00	91.48	244.45	−16.998634
γ-orth	$\sqrt{2}\times\sqrt{2}\times2$	9.03	8.78	12.44	90.00	90.00	90.00	246.67	−16.996339
δ-orth	$1\times1\times1$	4.83	10.64	18.07	90.00	90.00	90.00	232.26	−17.100486

Table S3: Local parameters of CsPbI bulk perovskites. Average effective coordination number on Pb atom (ECN_{av}^{Pb}), average Pb – I – Pb angles (θ_{av}^{PbIPb}) and average of distance between Pb and I (d_{av}^{PbI}).

Phase	Supercell	ECN_{av}^{Pb} (NNN)	θ_{av}^{PbIPb} (°)	d_{av}^{PbI} (Å)
α-cub	$1\times1\times1$	6.00	180.00	3.16
α-cub	$2\times2\times2$	6.00	180.00	3.16
α-orth	$2\times2\times2$	6.00	157.55	3.19
α-tric	$2\times2\times2$	6.00	154.91	3.21
γ-orth	$\sqrt{2}\times\sqrt{2}\times2$	6.00	161.30	3.18
δ-orth	$1\times1\times1$	5.79	93.20	3.27

Table S4: Electronic band gaps (E_g) of α, γ and δ phases of CsPbI-based perovskites measured by different experimental methods reported.

Phase	E_g (eV)	Method	Ref.
α	1.73	Photoluminicence	S1
	1.79	Photoluminicence	S2
	1.74	Photoluminicence	S3
	1.73	Photoluminicence	S4
	1.77	Absorbance	S5
	1.73	Absorbance	S6
	1.73	Absorbance	S7
	1.73	Absorbance	S8
	1.69	Absorbance	S9
	1.72	Absorbance/Photoluminicence	S10
average:	1.73		
γ	1.75	Absorbance/Photoluminicence	S11
	1.69	Absorbance/Photoluminicence	S10
	1.75	Absorbance/Photoluminicence	S12
	1.77	Spectroscopic ellipsometry	S13
average:	1.74		
δ	2.82	Photoluminicence	S14
	2.95	Absorbance	S7
	2.81	Absorbance	S9
	2.82	Absorbance/Photoluminicence	S10
	2.80	Absorbance/Photoluminicence	S12
	2.90	Spectroscopic ellipsometry	S13
average:	2.85		

Table S5: Rigid shift of the band gap calculated by means of the hybrid functional HSE (χ^{HSE}) and using the irreducible k-points of the k-mesh of $4 \times 4 \times 4$ for the α-CsPbI$_3$-$(1 \times 1 \times 1)$ bulk perovskite. The specific mixing coefficient of exact exchange (α_{XX}) used in Hartree–Fock calculations is also indicated. Since we have direct band gap, the band gap rigid shift on $\mathbf{k}_F$-point is indicated in boldface text.

$\mathbf{k}_i$-point (k_x, k_y, k_z)	$\chi^{HSE}_{\alpha_{XX}=0.25}$ (eV)	$\chi^{HSE}_{\alpha_{XX}=0.50}$ (eV)
0.00, 0.00, 0.00	0.61	1.25
0.25, 0.00, 0.00	0.62	1.27
0.50, 0.00, 0.00	0.53	1.12
0.25, 0.25, 0.00	0.64	1.31
0.50, 0.25, 0.00	0.52	1.10
0.50, 0.50, 0.00	0.51	1.08
0.25, 0.25, 0.25	0.61	1.27
0.50, 0.25, 0.25	0.50	1.07
0.50, 0.50, 0.25	0.50	1.06
**0.50, 0.50, 0.50**	**0.49**	**1.05**

Table S6: Rigid shift of the band gap calculated by means of the hybrid functional HSE (χ^{HSE}) and using the irreducible k-points of the k-mesh of $3 \times 3 \times 2$ for the γ-CsPbI$_3$-($\sqrt{2} \times \sqrt{2} \times 2$) bulk perovskite. The specific mixing coefficient of exact exchange (α_{XX}) used in Hartree–Fock calculations is also indicated. Since we have direct band gap, the band gap rigid shift on k$_F$-point is indicated in boldface text.

$\mathbf{k}_i$-point (k_x, k_y, k_z)	$\chi^{HSE}_{\alpha_{XX}=0.25}$ (eV)	$\chi^{HSE}_{\alpha_{XX}=0.50}$ (eV)
**0.00, 0.00, 0.00**	**0.50**	**1.05**
$0.3\bar{3}, 0.00, 0.00$	0.52	1.10
$0.00, 0.3\bar{3}, 0.00$	0.52	1.10
$0.3\bar{3}, 0.3\bar{3}, 0.00$	0.54	1.13
0.00, 0.00, 0.50	0.50	1.06
$0.3\bar{3}, 0.00, 0.50$	0.56	1.16
$0.00, 0.3\bar{3}, 0.50$	0.56	1.17
$0.3\bar{3}, 0.3\bar{3}, 0.50$	0.55	1.15

Table S7: Rigid shift of the band gap calculated by means of the hybrid functional HSE (χ^{HSE}) and using the irreducible k-points of the k-mesh of $5 \times 2 \times 1$ for the δ-CsPbI$_3$-($1 \times 1 \times 1$) bulk perovskite. The specific mixing coefficient of exact exchange (α_{XX}) used in Hartree–Fock calculations is also indicated. Since we have direct band gap, the band gap rigid shift on k$_F$-point is indicated in boldface text.

$\mathbf{k}_i$-point (k_x, k_y, k_z)	$\chi^{HSE}_{\alpha_{XX}=0.25}$ (eV)	$\chi^{HSE}_{\alpha_{XX}=0.50}$ (eV)
0.0, 0.00, 0.0	0.71	1.48
0.2, 0.00, 0.0	0.74	1.53
0.4, 0.00, 0.0	0.75	1.54
**0.0, 0.50, 0.0**	**0.72**	**1.48**
0.2, 0.50, 0.0	0.75	1.55
0.4, 0.50, 0.0	0.75	1.55

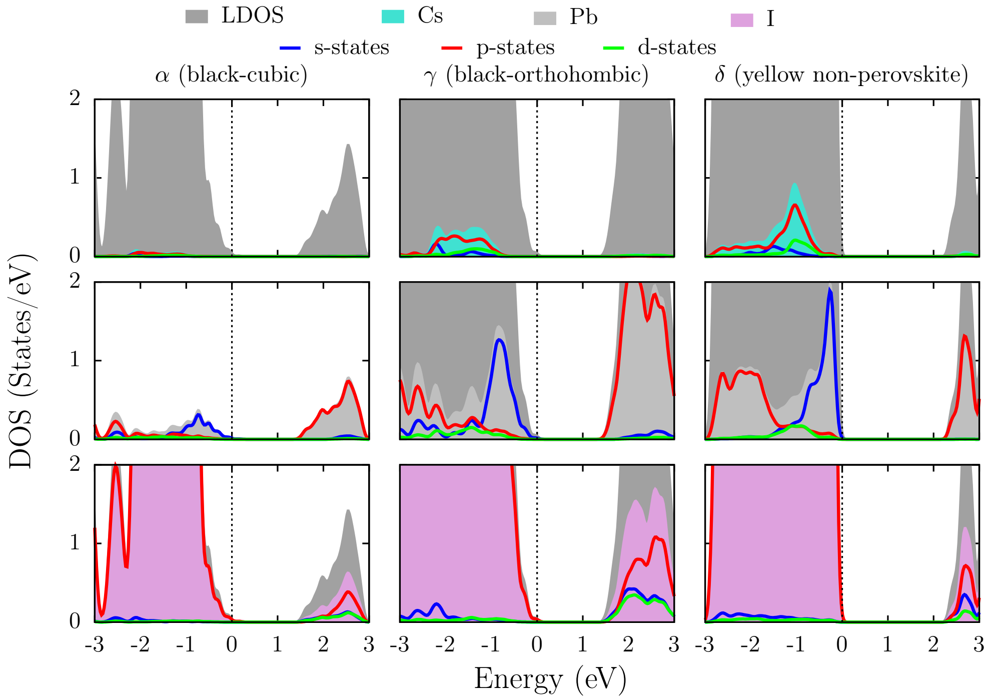

Figure S2: Local density of states and its decomposition per atomic specie and orbital for α-CsPbI$_3$-$(1\times1\times1)$, γ-CsPbI$_3$-$(\sqrt{2}\times\sqrt{2}\times2)$, and δ-CsPbI$_3$-$(1\times1\times1)$ bulk perovskites.

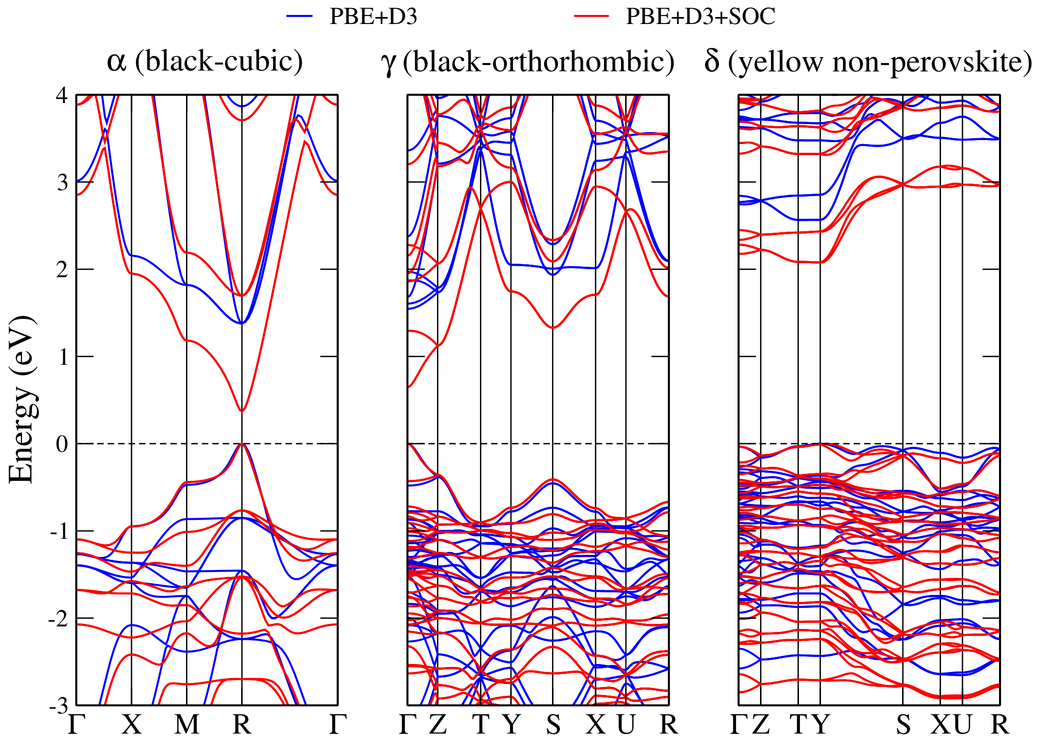

Figure S3: Electronic band structure for α-CsPbI$_3$-$(1\times1\times1)$, γ-CsPbI$_3$-$(\sqrt{2}\times\sqrt{2}\times2)$, and δ-CsPbI$_3$-$(1\times1\times1)$ bulk perovskites using the PBE+D3 and PBE+SOC methods.

Table S8: Effective Bader charge analysis for α-$CsPbI_3$-$(1\times1\times1)$, γ-$CsPbI_3$-$(\sqrt{2}\times\sqrt{2}\times2)$, and δ-$CsPbI_3$-$(1\times1\times1)$ bulk perovskites. $\bar{Q}_i$ is the average of effective Bader charge for species i = Cs, Pb, and I calculated from the difference between the number of valence electrons and the Bader charge at a given atom. The last column presents the net charge calculated from the formula unit stoichiometry. All effective charges are reported in units of electron charge (e).

Phase	$\bar{Q}_{Cs}$	$\bar{Q}_{Pb}$	$\bar{Q}_{I}$	$\sum_i Q_i$
α	0.87	0.90	−0.59	0.00
γ	0.85	0.93	−0.59	0.00
δ	0.82	0.94	−0.59	0.00

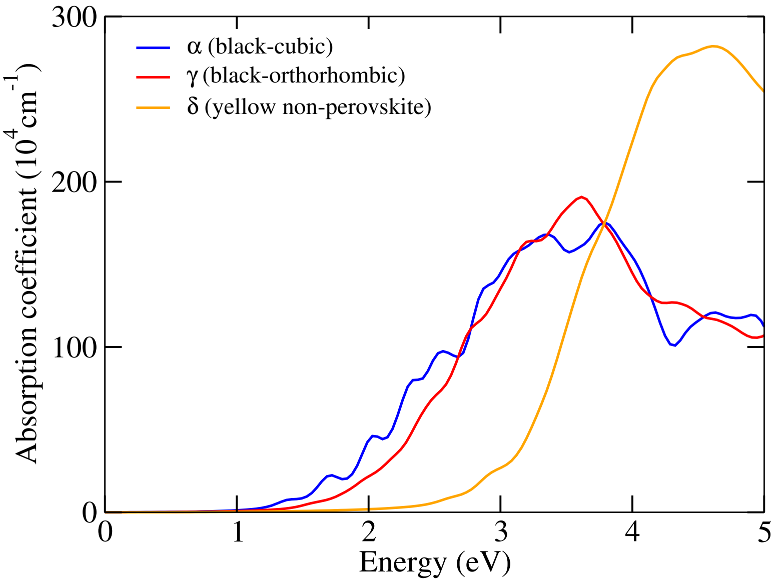

Figure S4: Sum of the absorption coefficients in the x, y and z directions for α-$CsPbI_3$-$(1\times1\times1)$, γ-$CsPbI_3$-$(\sqrt{2}\times\sqrt{2}\times2)$, and δ-$CsPbI_3$-$(1\times1\times1)$ bulk perovskites calculated by using the PBE+D3 method.

3 Molecules in Gas Phase

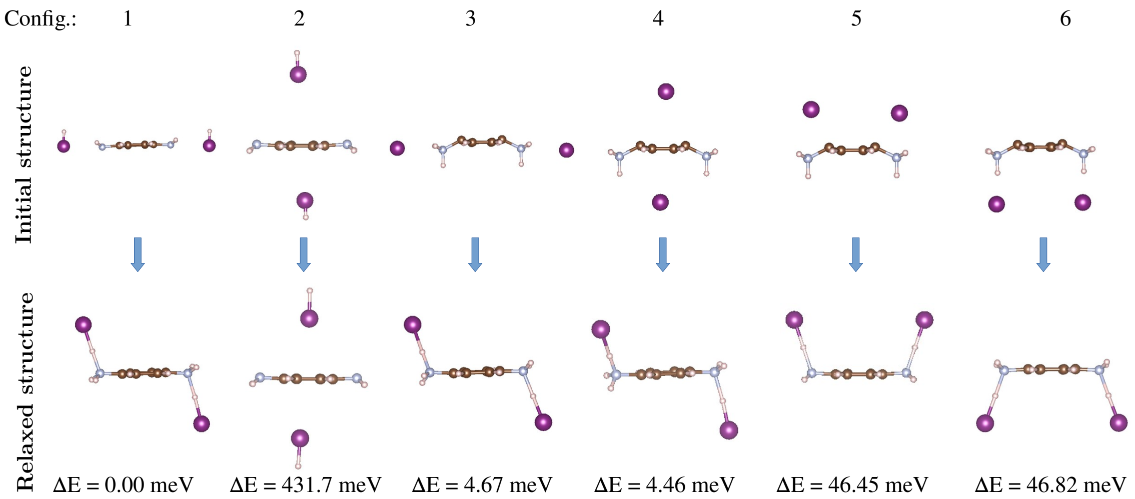

Figure S5: PPDI molecules in gas phase. Upper panels show the initial geometries and the bottom panels show the optimized geometries.

Table S9: Energetic properties of PPD and PPDI molecules. Total energy (E_{tot}), relative energy for PPDI molecules (ΔE_{tot}), cohesive energy per atom (E_{coh}), gap HOMO-LUMO (Δ^{H-L}) and ionization potential (Φ).

Molecule	Config.	E_{tot} (eV)	ΔE_{tot} (meV)	E_{coh} (eV/atom)	$\Delta^{HOMO-LUMO}$ (eV)	Φ (eV)
PPD	-	-102.891055	-	-4.28	0.53	2.23
PPD^{+2}	-	-91.161528	-	-	4.89	-
PPDI	1	-110.800734	0.00	-4.23	3.39	5.60
	2	-110.372888	431.70	-4.21	2.90	4.46
	3	-110.794446	4.67	-4.23	3.00	5.32
	4	-110.796214	4.46	-4.23	2.79	5.19
	5	-110.753159	46.45	-4.22	3.52	5.89
	6	-110.760360	46.82	-4.22	3.52	5.90

Table S10: Nearest distances between H and N atoms ($d^{\mathrm{N-H}}$) and H and I atoms ($d^{\mathrm{H-I}}$) of the two NH_3 groups of PPD, PPD^{2+} and PPDI molecules. All values are reported in Å.

Molecule	Config.	$d_1^{\mathrm{N-H}}$	$d_2^{\mathrm{N-H}}$	$d_3^{\mathrm{N-H}}$	$d^{\mathrm{H-I}}$
PPD	-	1.10	1.03	1.03	-
		1.10	1.03	1.03	-
PPD^{+2}	-	1.04	1.04	1.04	-
		1.04	1.04	1.04	-
PPDI	1	1.54	1.02	1.02	1.76
		1.58	1.02	1.02	1.75
	2	-	1.02	1.02	1.63
		-	1.02	1.02	1.63
	3	1.36	1.02	1.02	1.86
		1.64	1.02	1.02	1.73
	4	1.29	1.02	1.03	1.92
		1.70	1.02	1.02	1.71
	5	1.61	1.02	1.02	1.74
		1.61	1.02	1.02	1.74
	6	1.60	1.02	1.02	1.74
		1.61	1.02	1.02	1.74

Table S11: Effective Bader charge analysis of PPD molecule in gas phase. Q is the Bader charge, Z_{val} is the number of valence electrons and $Q_{eff} = Z_{val} - Q$ is the effective Bader charge. The atoms that make up the NH_3 groups are indicated in boldface. All values are reported in units of electron charge (e).

Molecule	Atom	Q	Z_{val}	Q_{eff}
PPD	C	4.04	4	−0.04
	C	4.05	4	−0.05
	C	3.90	4	0.10
	C	4.06	4	−0.06
	C	4.06	4	−0.06
	C	3.91	4	0.09
	H	1.01	1	−0.01
	H	1.02	1	−0.02
	H	1.00	1	0.00
	H	1.01	1	−0.01
	**H**	**0.55**	**1**	**0.45**
	**H**	**0.55**	**1**	**0.45**
	**H**	**0.55**	**1**	**0.45**
	**H**	**0.55**	**1**	**0.45**
	**H**	**0.78**	**1**	**0.22**
	**H**	**0.78**	**1**	**0.22**
	**N**	**6.08**	**5**	**− 1.08**
	**N**	**6.09**	**5**	**− 1.09**

Table S12: Effective Bader charge analysis of PPD^{2+} molecule in gas phase. Q is the Bader charge, Z_{val} is the number of valence electrons and $Q_{eff} = Z_{val} - Q$ is the effective Bader charge. The atoms that make up the NH_3 groups are indicated in boldface. All values are reported in units of electron charge (e).

Molecule	Atom	Q	Z_{val}	Q_{eff}
PPD^{2+}	C	3.98	4	0.02
	C	3.94	4	0.06
	C	3.82	4	0.18
	C	3.97	4	0.03
	C	3.94	4	0.06
	C	3.82	4	0.18
	H	0.87	1	0.13
	H	0.87	1	0.13
	H	0.88	1	0.12
	H	0.87	1	0.13
	**H**	**0.47**	**1**	**0.53**
	**H**	**0.46**	**1**	**0.54**
	**H**	**0.47**	**1**	**0.53**
	**H**	**0.47**	**1**	**0.53**
	**H**	**0.47**	**1**	**0.53**
	**H**	**0.47**	**1**	**0.53**
	**N**	**6.12**	**5**	**– 1.12**
	**N**	**6.11**	**5**	**– 1.11**

Table S13: Effective Bader charge analysis of PPDI putative global minima configuration (PGMC) molecule in gas phase. Q is the Bader charge, Z_{val} is the number of valence electrons and $Q_{eff} = Z_{val} - Q$ is the effective Bader charge. The I atoms and those forming the NH_3 groups are indicated in boldface. All values are reported in units of electron charge (e).

Molecule	Atom	Q	Z_{val}	Q_{eff}
PPDI (PGMC)	C	4.02	4	−0.02
	C	3.97	4	0.03
	C	3.70	4	0.30
	C	3.99	4	0.01
	C	3.99	4	0.01
	C	3.70	4	0.30
	H	0.95	1	0.05
	H	0.96	1	0.04
	H	0.96	1	0.04
	H	0.96	1	0.04
	**H**	**0.56**	**1**	**0.44**
	**H**	**0.55**	**1**	**0.45**
	**H**	**0.55**	**1**	**0.45**
	**H**	**0.55**	**1**	**0.45**
	**H**	**0.80**	**1**	**0.20**
	**H**	**0.84**	**1**	**0.16**
	**N**	**6.13**	**5**	**−1.13**
	**N**	**6.13**	**5**	**−1.13**
	**I**	**7.32**	**7**	**−0.32**
	**I**	**7.38**	**7**	**−0.38**

Table S14: Effective Bader charge analysis of PPDI with higher energy configuration (HEC) molecule in gas phase. Q is the Bader charge, Z_{val} is the number of valence electrons and $Q_{eff} = Z_{val} - Q$ is the effective Bader charge. The I atoms and those forming the NH_3 groups are indicated in boldface. All values are reported in units of electron charge (e).

Molecule	Atom	Q	Z_{val}	Q_{eff}
PPDI (HEC)	C	4.01	4	−0.01
	C	4.00	4	0.00
	C	3.70	4	0.30
	C	4.01	4	−0.01
	C	3.99	4	0.01
	C	3.68	4	0.32
	H	0.95	1	0.05
	H	0.96	1	0.04
	H	0.96	1	0.04
	H	0.95	1	0.05
	**H**	**0.56**	**1**	**0.44**
	**H**	**0.56**	**1**	**0.44**
	**H**	**0.55**	**1**	**0.45**
	**H**	**0.55**	**1**	**0.45**
	**H**	**0.85**	**1**	**0.15**
	**H**	**0.85**	**1**	**0.15**
	**N**	**5.76**	**5**	**−1.13**
	**N**	**5.91**	**5**	**−1.15**
	**I**	**6.79**	**7**	**−0.29**
	**I**	**6.96**	**7**	**−0.29**

4 Passivated Surfaces

4.1 Total and relative energies

Table S15: Rearrangement following the minimum energy of the several configuration of the PPD/α-$CsPbI_3(100)$-(2×1), PPD/$\alpha - CsPbI_3(100) - (\sqrt{2}\times\sqrt{2})$, PPD/$\gamma - CsPbI_3(100) - (\sqrt{2}\times\sqrt{2})$, PPD/$\delta$-$CsPbI_3(100)$-$(1\times1)$ and PPD/δ_F-$CsPbI_3(100)$-(1×1) PPD passivated layers.

Config.	$\alpha-(2\times1)$	α-$(\sqrt{2}\times\sqrt{2})$	γ-$(\sqrt{2}\times\sqrt{2})$	δ-(1×1)	δ_F-(1×1)
1	r5	h4	h2	r2	r3
2	r1	h1	h3	h1	h1
3	h2	r1	r6	r1	r2
4	r4	h2	r4	r4	r5
5	h3	h3	r3	h4	r1
6	r6	r5	h4	h2	r4
7	r2	r6	h1	r3	h2
8	r3	r3	r2	r5	h3
9	h1	r4	r5	h3	h4
10	h4	r2	r1	h5	h5

Table S16: Total energy of the several configuration of the x/α**-**$CsPbI_3(100)$**-**$(2{\times}1)$**,** $x/\alpha-CsPbI_3(100)-(\sqrt{2}\times\sqrt{2})$**,** $x/\gamma-CsPbI_3(100)-(\sqrt{2}\times\sqrt{2})$**,** $x/\delta-CsPbI_3(100)-(1{\times}1)$ **and** $x/\delta_F-CsPbI_3(100)-(1{\times}1)$ **PPD passivated layers, where** $x=$ **PPD or Cs. All values are reported in** eV**.**

Passivator	Config.	$\alpha-(2{\times}1)$	α-$(\sqrt{2}\times\sqrt{2})$	γ-$(\sqrt{2}\times\sqrt{2})$	δ-(1×1)	δ_F-(1×1)
PPD	1	−379.475559	−378.891256	−379.040137	−397.991362	−397.955819
	2	−379.258419	−378.773123	−378.602542	−397.503907	−397.736746
	3	−379.201504	−378.644223	−378.117851	−397.155263	−397.660750
	4	−379.143069	−378.500411	−377.869255	−397.111822	−397.381620
	5	−379.125546	−378.041324	−377.757548	−397.089039	−397.145420
	6	−377.853211	−377.721810	−377.708454	−396.937206	−397.142482
	7	−377.688743	−377.705147	−377.646200	−396.650904	−397.024869
	8	−377.687048	−377.622321	−377.568028	−396.562997	−396.360246
	9	−376.096247	−377.339273	−377.434404	−396.122590	−396.336260
	10	−373.979083	−377.111214	−375.029791	−395.841999	−395.902071
Cs	-	−183.800415	−184.017450	−184.166283	−202.425931	-

Table S17: Relative energy (ΔE**) of the several configuration of the PPD/**α**-**$CsPbI_3(100)$**-**$(2{\times}1)$**,** PPD$/\alpha-CsPbI_3(100)-(\sqrt{2}\times\sqrt{2})$**,** PPD$/\gamma-CsPbI_3(100)-(\sqrt{2}\times\sqrt{2})$**, PPD/**$\delta$**-**$CsPbI_3(100)$**-**$(1{\times}1)$ **and PPD/**δ_F**-**$CsPbI_3(100)$**-**$(1{\times}1)$ **PPD passivated layers. For the black phases, relative energies are quantified respect to the lowest value, which correspond to the configuration 1 for the PPD/**α**-**$CsPbI_3(100)$**-**$(2{\times}1)$ **layer. For the yellow phases, the energy reference correspond to the configuration 1 for the PPD/**δ**-**$CsPbI_3(100)$**-**$(1{\times}1)$ **layer. All values are reported in** eV**.**

Config.	$\alpha-(2{\times}1)$	α-$(\sqrt{2}\times\sqrt{2})$	γ-$(\sqrt{2}\times\sqrt{2})$	δ-(1×1)	δ_F-(1×1)
1	0.000000	0.584304	0.435423	0.000000	0.035544
2	0.217141	0.702437	0.873018	0.487455	0.254616
3	0.274055	0.831336	1.357709	0.836100	0.330613
4	0.332490	0.975148	1.606305	0.879540	0.609742
5	0.350013	1.434235	1.718011	0.902323	0.845943
6	1.622348	1.753750	1.767106	1.054157	0.848881
7	1.786816	1.770412	1.829359	1.340459	0.966494
8	1.788511	1.853238	1.907531	1.428366	1.631116
9	3.379313	2.136286	2.041155	1.868773	1.655102
10	5.496476	2.364346	4.445769	2.149363	2.089292

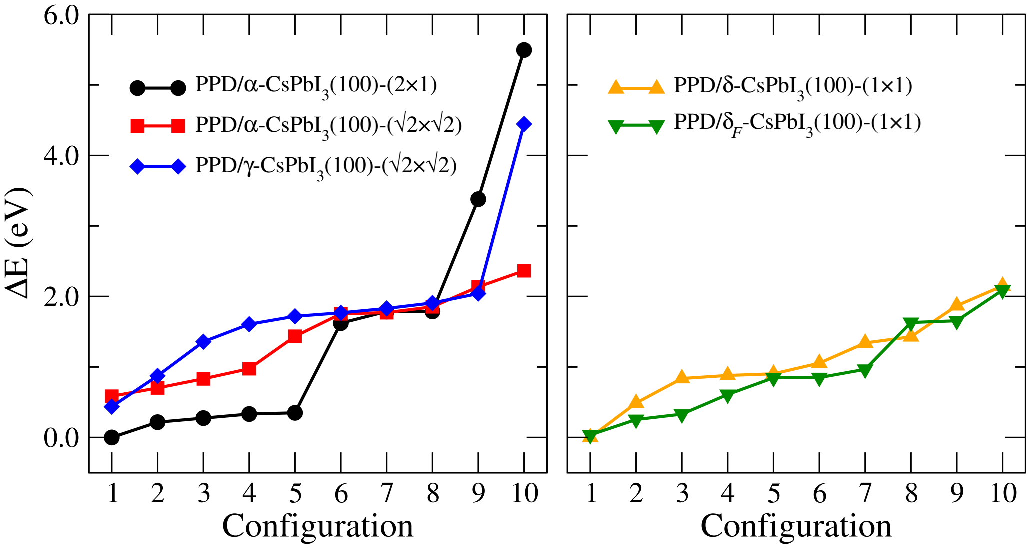

Figure S6: Relative energy (ΔE) as a function of the structure Configuration for the several configuration of the PPD/α-$CsPbI_3(100)$-(2×1), PPD/α-$CsPbI_3(100)$-$(\sqrt{2}\times\sqrt{2})$, PPD/γ-$CsPbI_3(100)$-$(\sqrt{2}\times\sqrt{2})$, PPD/δ-$CsPbI_3(100)$-(1×1) and PPD/δ_F-$CsPbI_3(100)$-(1×1) passivated layers. All black phases structures, adopt an unique reference as they have the same stoichiometry. Similarly, the two sets of yellow structures have adopt an unique reference.

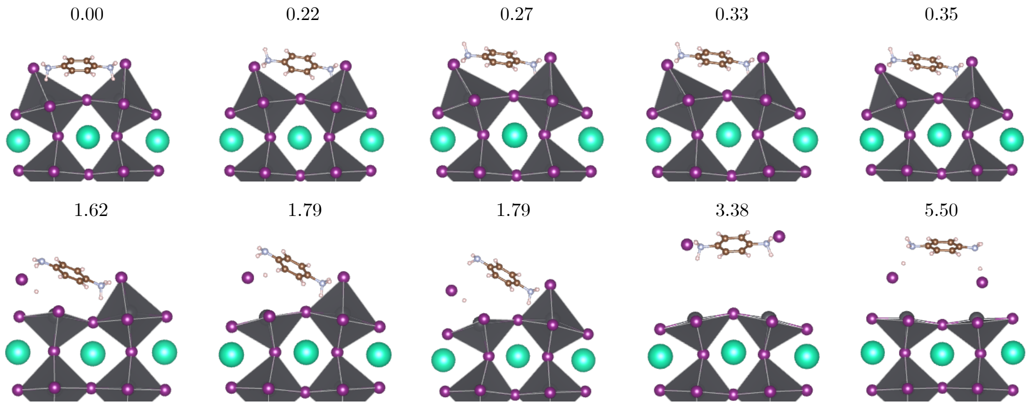

Figure S7: The ten relaxed configuration of the PPD/α-$CsPbI_3(100)$-(2×1) layers, ordered from lowest to highest energy. The numbers at the top of the crystal structures are the relative energies compared to the lowest energy black-phase material.

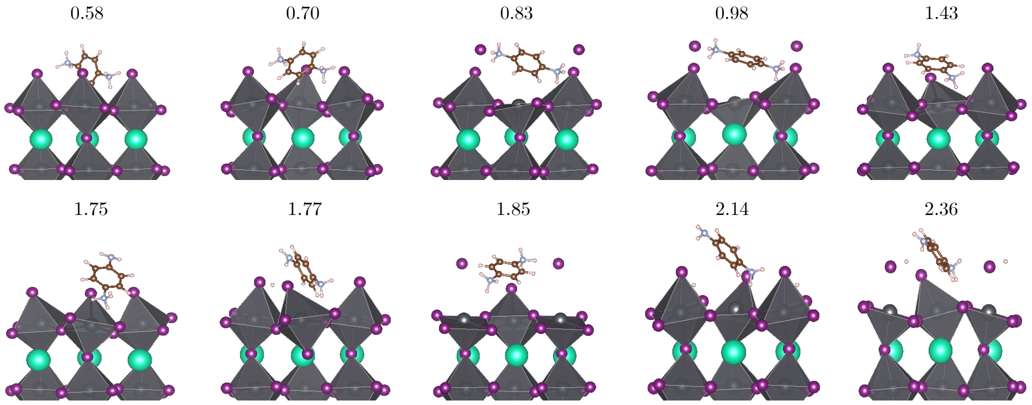

Figure S8: The ten relaxed configuration of the PPD/α-$\mathrm{CsPbI_3}(100)$-$(\sqrt{2}\times\sqrt{2})$ layers, ordered from lowest to highest energy. The numbers at the top of the crystal structures are the relative energies compared to the lowest energy black-phase material.

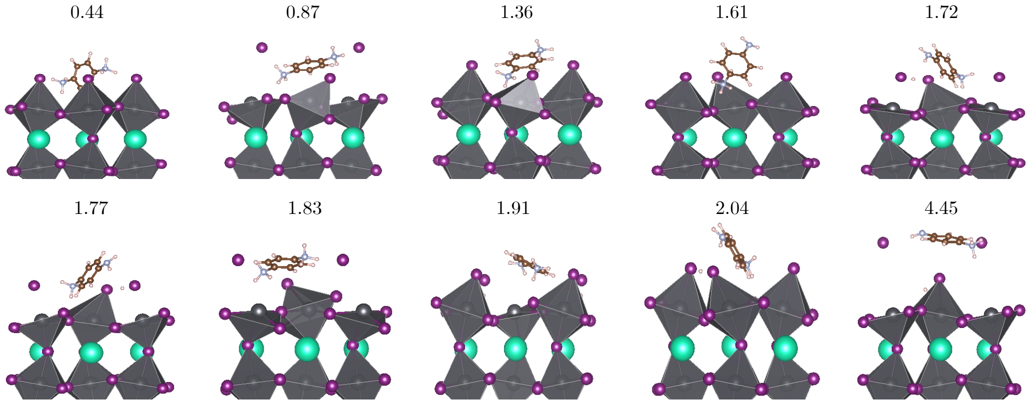

Figure S9: The ten relaxed configuration of the PPD/γ-$\mathrm{CsPbI_3}(100)$-$(\sqrt{2}\times\sqrt{2})$ layers, ordered from lowest to highest energy. The numbers at the top of the crystal structures are the relative energies compared to the lowest energy black-phase material.

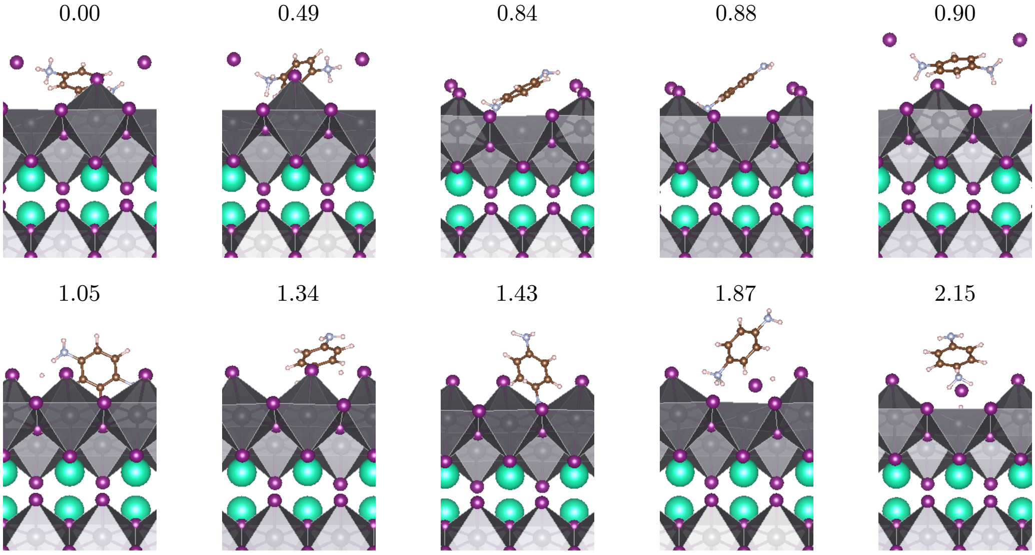

Figure S10: The ten relaxed configuration of the PPD/δ-$\mathrm{CsPbI_3}(100)$-(1×1) layers, ordered from lowest to highest energy. The numbers at the top of the crystal structures are the relative energies compared to the lowest energy yellow-phase material.

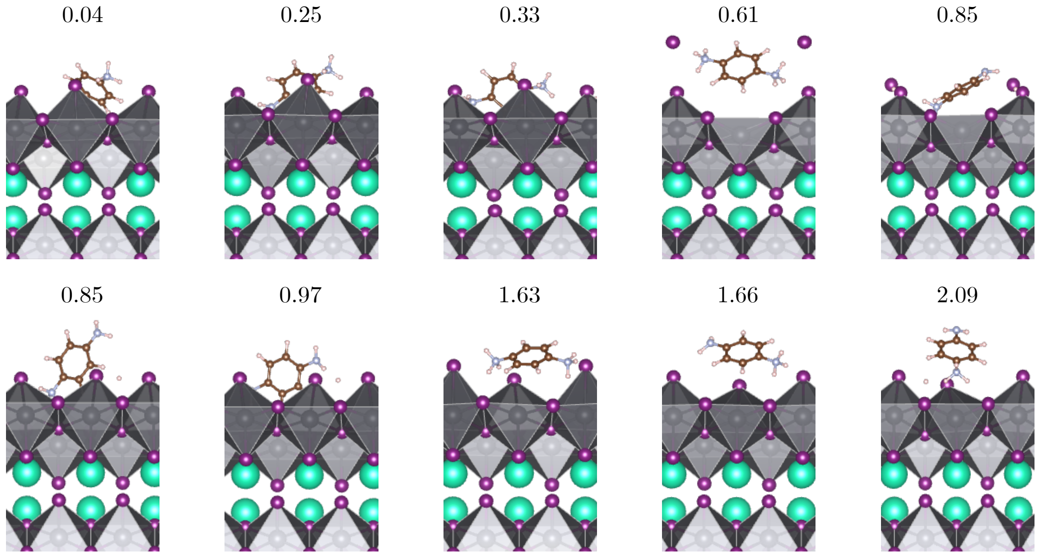

Figure S11: The ten relaxed configuration of the PPD/δ_F-$CsPbI_3(100)$-(1×1) frozen layers, ordered from lowest to highest energy. The numbers at the top of the crystal structures are the relative energies compared to the lowest energy yellow-phase material.

4.2 Surfaces formation energies

Table S18: Surface formation energy (E_F^S) of the several configuration of the x/α-$CsPbI_3(100)$-(2×1), x/α-$CsPbI_3(100)$-$(\sqrt{2}\times\sqrt{2})$, x/γ-$CsPbI_3(100)$-$(\sqrt{2}\times\sqrt{2})$, x/δ-$CsPbI_3(100)$-(2×1) and x/δ_F-$CsPbI_3(100)$-(2×1) passivated layers, where $x=$ PPD or Cs. All values are reported in meV/Å^2.

Passivator	Config.	$\alpha-(2\times1)$	$\alpha-(\sqrt{2}\times\sqrt{2})$	$\gamma-(1\times1)$	$\delta-(1\times1)$	$\delta_F-(1\times1)$
PPD	1	−24.91	−21.25	−19.46	−13.24	−13.06
	2	−23.55	−20.51	−16.71	−10.86	−12.00
	3	−23.19	−19.70	−13.65	−9.17	−11.63
	4	−22.82	−18.80	−12.08	−8.96	−10.27
	5	−22.72	−15.93	−11.38	−8.85	−9.12
	6	−14.75	−13.93	−11.07	−8.11	−9.11
	7	−13.72	−13.82	−10.68	−6.72	−8.54
	8	−13.71	−13.30	−10.18	−6.29	−5.30
	9	−3.75	−11.53	−9.34	−4.15	−5.19
	10	9.50	−10.10	5.81	−2.78	−3.08
Cs	-	4.74	3.38	6.06	13.36	-

4.3 Local parameters

Table S19: Octahedron distortions for the x/α-$CsPbI_3(100)$-(2×1), x/α-$CsPbI_3(100)$-$(\sqrt{2}\times\sqrt{2})$, x/γ-$CsPbI_3(100)$-$(\sqrt{2}\times\sqrt{2})$, x/δ-$CsPbI_3(100)$-(2×1) and x/δ_F-$CsPbI_3(100)$-(2×1) passivated layers, where x = PPD or Cs. Average angle I−Pb−I close to 90° ($\theta_{av}^{\mathrm{IPbI-90}}$), average angle I−Pb−I close to 180° ($\theta_{av}^{\mathrm{IPbI-180}}$) and average angle Pb−I−Pb ($\theta_{av}^{\mathrm{PbIPb}}$). All values are reported in °.

Phase	$\theta_{av}^{\mathrm{IPbI-90}}$	$\theta_{av}^{\mathrm{IPbI-180}}$	$\theta_{av}^{\mathrm{PbIPb}}$
Cs/α-$CsPbI_3(100)$-(2×1)	89.92	176.01	176.15
PPD/α-$CsPbI_3(100)$-(2×1)	89.89	172.11	169.26
Cs/α-$CsPbI_3(100)$-$(\sqrt{2}\times\sqrt{2})$	89.93	176.62	176.74
PPD/α-$CsPbI_3(100)$-$(\sqrt{2}\times\sqrt{2})$	89.98	175.91	166.42
Cs/γ-$CsPbI_3(100)$-$(\sqrt{2}\times\sqrt{2})$	89.96	176.86	163.06
PPD/γ-$CsPbI_3(100)$-$(\sqrt{2}\times\sqrt{2})$	90.00	175.47	159.57
Cs/δ-$CsPbI_3(100)$-(1×1)	89.94	173.10	92.38
PPD/δ-$CsPbI_3(100)$-(1×1)	90.21	173.61	93.07
PPD/δ_F-$CsPbI_3(100)$-(1×1)	90.00	170.67	94.01

4.4 Electronic density of states

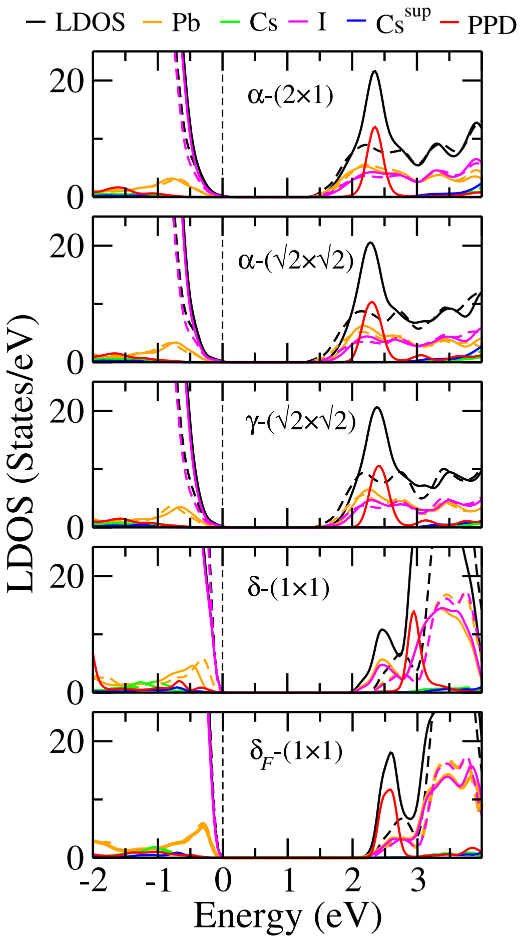

Figure S12: Local density of states for the x/α-CsPbI$_3$(100)-(2×1), x/α-CsPbI$_3$(100)-($\sqrt{2}\times\sqrt{2}$), x/γ-CsPbI$_3$(100)-($\sqrt{2}\times\sqrt{2}$), x/δ-CsPbI$_3$(100)-(2×1) and x/δ_F-CsPbI$_3$(100)-(2×1) passivated layers, where x = PPD or Cs. The dashed lines indicate the case of passivation with Cs. The calculations were performed by using the PBE+D3 method.

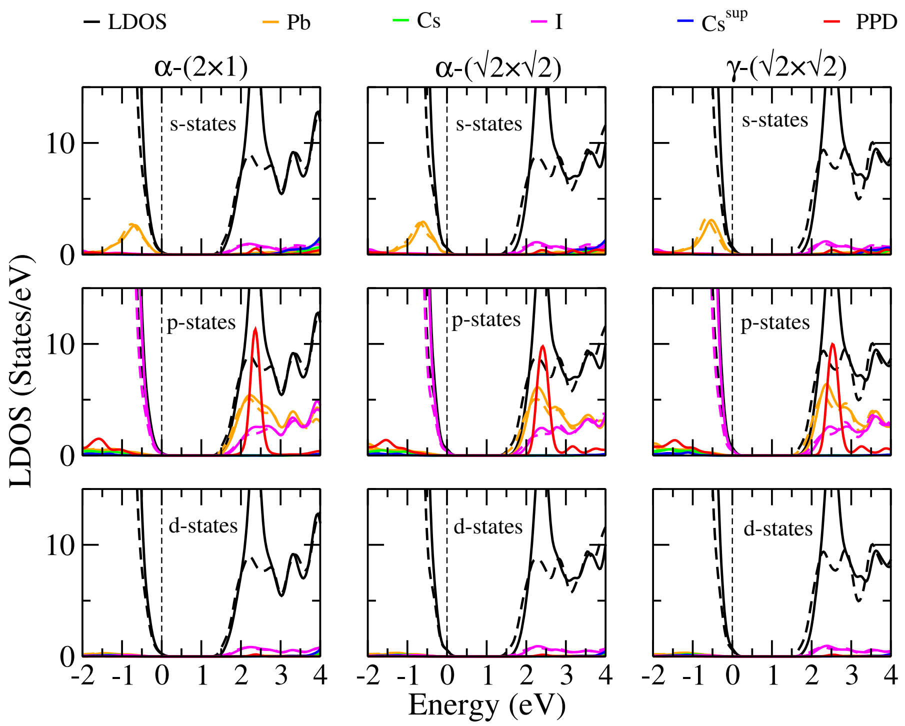

Figure S13: Local density of states and their projection on orbitals s, p and d for the x/α-CsPbI$_3$(100)-(2×1), x/α-CsPbI$_3$(100)-($\sqrt{2}\times\sqrt{2}$) and x/γ-CsPbI$_3$(100)-($\sqrt{2}\times\sqrt{2}$) passivated layers, where x = PPD or Cs. The dashed lines indicate the case of passivation with Cs. The Fermi level is set to zero. The calculations were performed by using the PBE+D3 method.

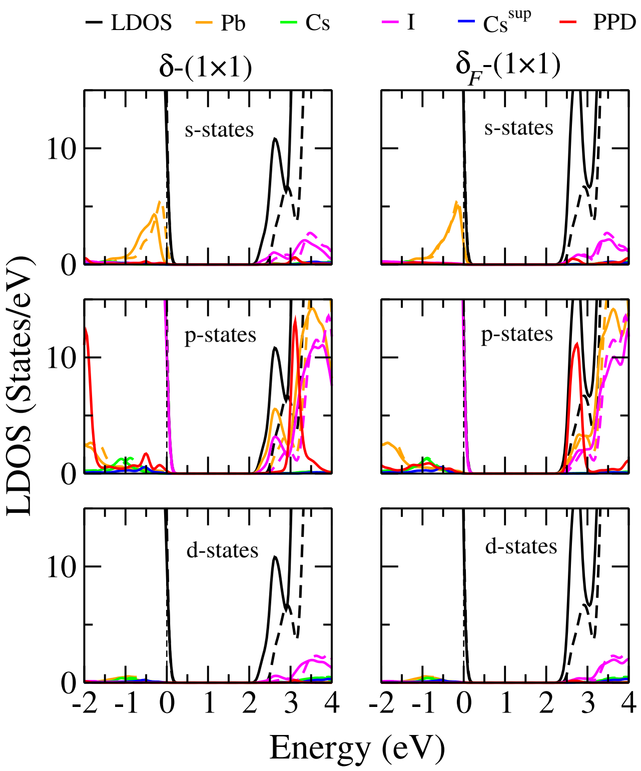

Figure S14: Local density of states and their projection on orbitals s, p and d for the x/δ-CsPbI$_3$(100)-(2×1) and x/δ_F-CsPbI$_3$(100)-(2×1). The dashed lines indicate the case of passivation with Cs. The Fermi level is set to zero. The calculations were performed by using the PBE+D3 method.

4.5 Electronic band structure

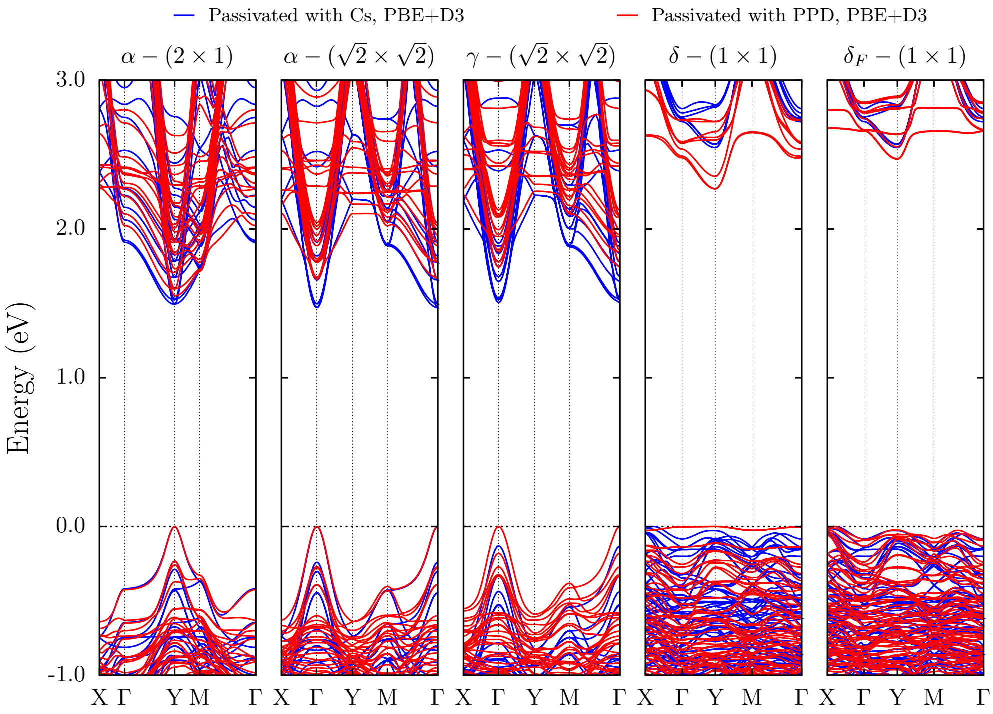

Figure S15: Electronic band structure for the x/α-$CsPbI_3(100)$-(2×1), x/α-$CsPbI_3(100)$-$(\sqrt{2}\times\sqrt{2})$, x/γ-$CsPbI_3(100)$-$(\sqrt{2}\times\sqrt{2})$, x/δ-$CsPbI_3(100)$-(2×1) and x/δ_F-$CsPbI_3(100)$-(2×1) passivated layers, where x = PPD or Cs. The Fermi level is set to zero. All band structure have been calculated by using the PBE+D3 method.

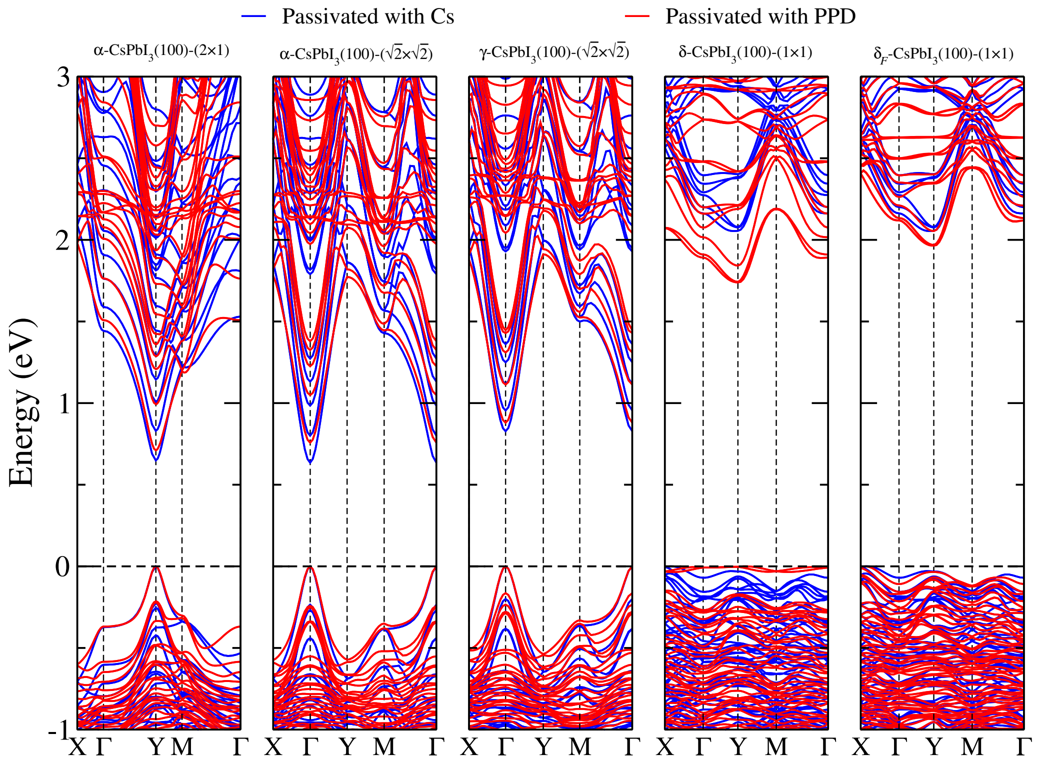

Figure S16: Electronic band structure for the x/α-CsPbI$_3$(100)-(2×1), x/α-CsPbI$_3$(100)-($\sqrt{2}\times\sqrt{2}$), x/γ-CsPbI$_3$(100)-($\sqrt{2}\times\sqrt{2}$), x/δ-CsPbI$_3$(100)-(2×1) and x/δ_F-CsPbI$_3$(100)-(2×1) passivated layers, where x = PPD or Cs. The Fermi level is set to zero. All band structure have been calculated by using the PBE+D3+SOC method.

4.6 Quantum Confinement

In this section we will calculate the change in the band gap values due to the quantum confinement, namely, $\chi^{\mathrm{QC}}(\mathrm{Cs}) = E_g^{\mathrm{PBE+D3+SOC}}(B) - E_g^{\mathrm{PBE+D3+SOC}}(S_{\mathrm{Cs}})$ and $\chi^{\mathrm{QC}}(\mathrm{PPD}) = E_g^{\mathrm{PBE+D3+SOC}}(B_{\mathrm{PPD}}) - E_g^{\mathrm{PBE+D3+SOC}}(S_{\mathrm{PPD}})$, for and Cs/$\alpha$-CsPbI$_3$(100)-(2×1) and PPD/$\alpha$-CsPbI$_3$(100)-(2×1) cases, respectively.

For the Cs passivation case, we know all band gaps values,
$E_g^{\mathrm{PBE+D3+SOC}}(B) = 0.37\,\mathrm{eV}$ and $E_g^{\mathrm{PBE+D3+SOC}}(S_{\mathrm{Cs}}) = 0.64\,\mathrm{eV}$, then, $\chi^{\mathrm{QC}}(\mathrm{Cs}) = -0.27\,\mathrm{eV}$.

On the other hand, for PPD passivation case, we only have the value of $E_g^{\mathrm{PBE+D3+SOC}}(S_{\mathrm{PPD}}) = 0.71\,\mathrm{eV}$. In Table 1 and Figure 1, we show the values of $E_g^{\mathrm{PBE+D3+SOC}}(S_{\mathrm{PPD}})$ when the thickness of the slab increases. From these values we found the following analytic expression $E_g^{\mathrm{PBE+D3+SOC}}(S_{\mathrm{PPD}}(n)) = 0.36e^{-0.344(n-4)} + 0.445$, where n is the number of the PbI$_6$ inner octahedra layers of the slab. Taking the limite case when n tends to infinity $E_g^{\mathrm{PBE+D3+SOC}}(S_{\mathrm{PPD}}(n \to \infty)) = 0.445\,\mathrm{eV}$, which cor-

respond to the band gap value of $E_g^{\text{PBE+D3+SOC}}(B_{\text{PPD}})$. Thus, $\chi^{\text{QC}}(\text{PPD}) = -0.27\,\text{eV}$. Therefore, $\chi^{\text{QC}}(\text{PPD}) = \chi^{\text{QC}}(\text{Cs})$, that means the change on the band gap values due to quantum confinement does not depends on the nature of the passivator. For the other phases, we use the same methodology and whose band gap values are shown in Table 4 in the manuscript.

Table S20: The band gap ($E_g^{\text{PBE+D3+SOC}}$) values of the PPD/α-CsPbI$_3$(100)-(2×1) versus the increments of the number of PbI$_6$ inner octahedra layers (n) and its corresponding values calculated using the equation $E_g^{\text{PBE+D3+SOC}}(S_{\text{PPD}}(n)) = 0.36e^{-0.344(n-4)} + 0.445$.

n	$E_g^{\text{PBE+D3+SOC}}(S_{\text{PPD}})$ (eV)	$E_g^{\text{PBE+D3+SOC}}(S_{\text{PPD}}(n))$ (eV)
5	0.711	0.712
6	0.633	0.634
7	0.579	0.579
8	0.539	0.540

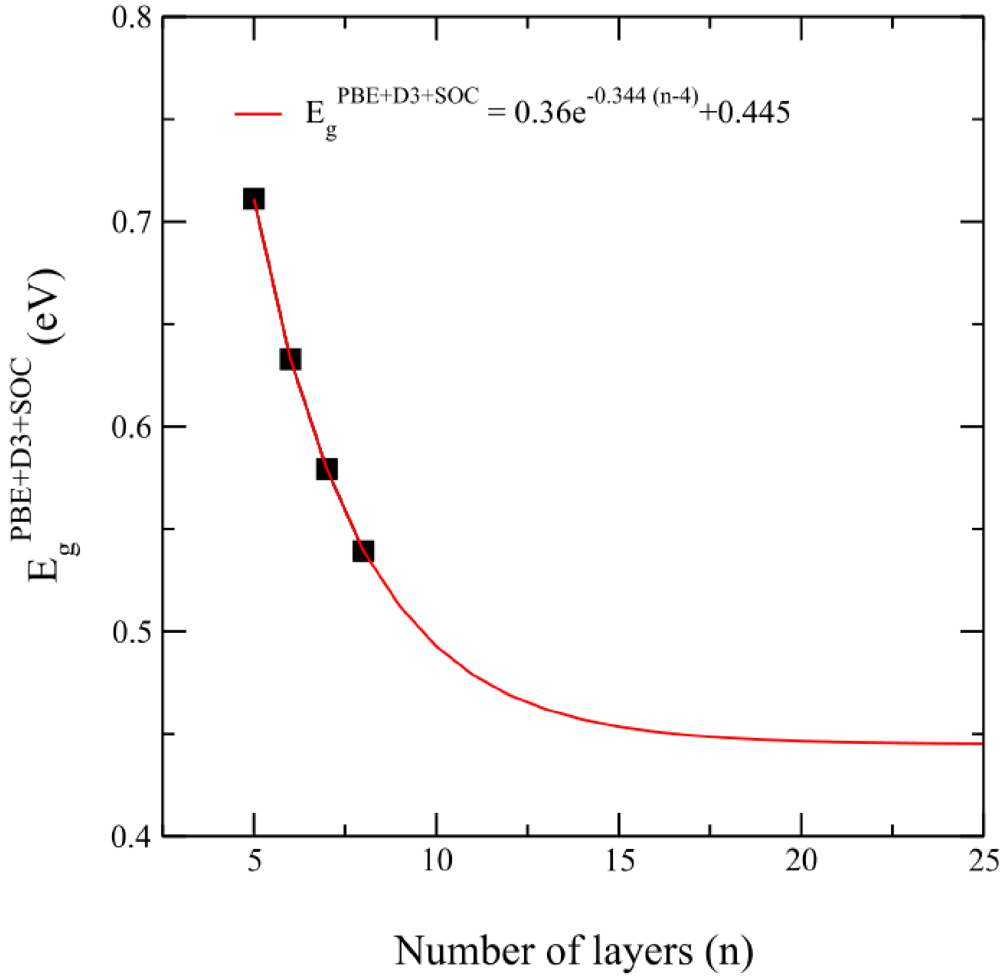

Figure S17: The band gap ($E_g^{\text{PBE+D3+SOC}}$) variation originated by increments of the thickness in the PPD/α-CsPbI$_3$(100)-(2×1).

4.7 Absorption coefficients

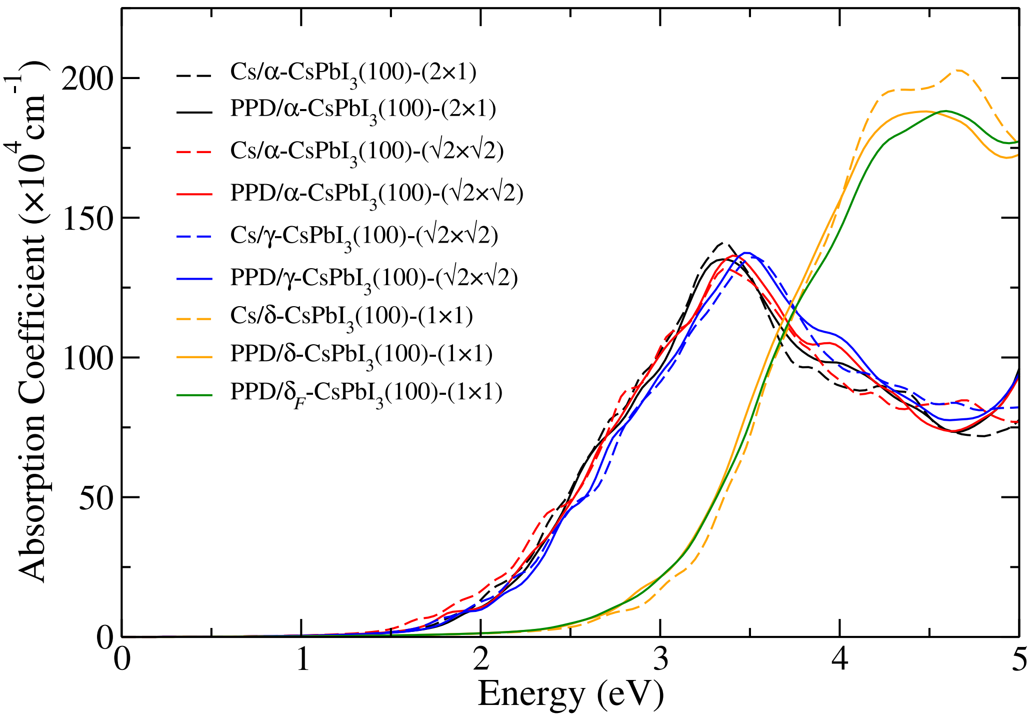

Figure S18: Absorption coefficient calculated using the PBE+D3 method for the x/α-CsPbI3(100)-(2×1), x/α-CsPbI3(100)-($\sqrt{2}\times\sqrt{2}$), x/γ-CsPbI3(100)-($\sqrt{2}\times\sqrt{2}$), x/δ-CsPbI3(100)-(2×1) and x/δ_F-CsPbI3(100)-(2×1) passivated layers, where x = PPD or Cs.

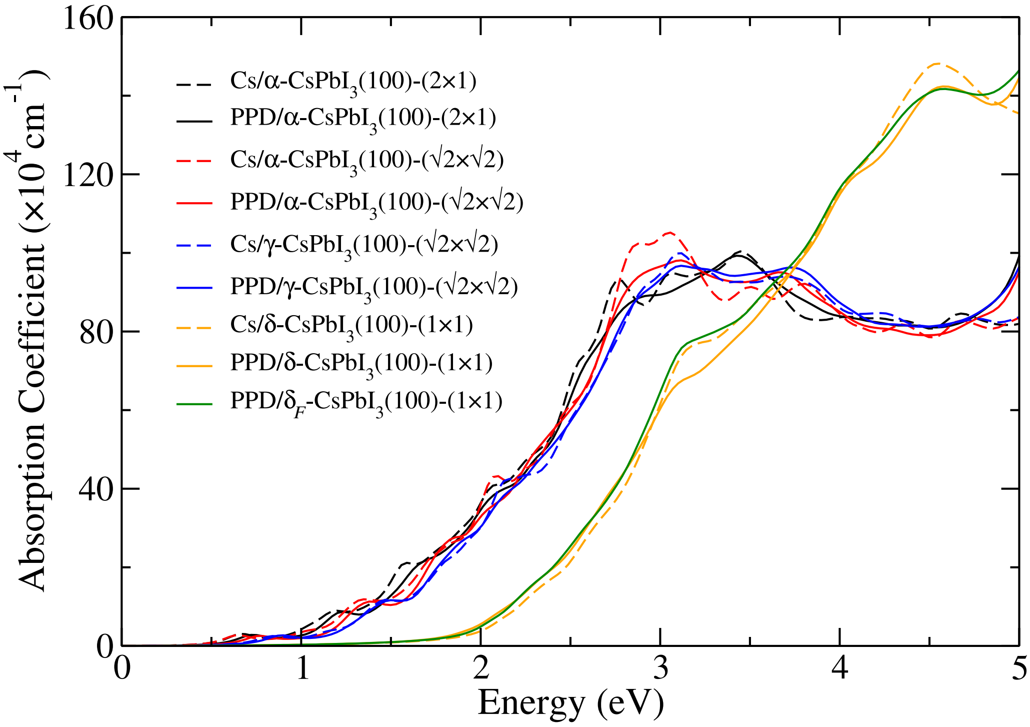

Figure S19: Absorption coefficient calculated using the PBE+D3+SOC method for the x/α-CsPbI3(100)-(2×1), x/α-CsPbI3(100)-($\sqrt{2}\times\sqrt{2}$), x/γ-CsPbI3(100)-($\sqrt{2}\times\sqrt{2}$), x/δ-CsPbI3(100)-(2×1) and x/δ_F-CsPbI3(100)-(2×1) passivated layers, where x = PPD or Cs.

References

(S1) Garza, A. J.; Scuseria, G. E. Predicting Band Gaps with Hybrid Density Functionals. *J. Phys. Chem. Lett.* **2016**, *7*, 4165–4170.

(S2) Zhou, W.; Sui, F.; Zhong, G.; Cheng, G.; Pan, M.; Yang, C.; Ruan, S. Lattice Dynamics and Thermal Stability of Cubic-Phase $CsPbI_3$ Quantum Dots. *J. Phys. Chem. Lett.* **2018**, *9*, 4915–4920, PMID: 30107128.

(S3) Protesescu, L.; Yakunin, S.; Bodnarchuk, M. I.; Krieg, F.; Caputo, R.; Hendon, C. H.; Yang, R. X.; Walsh, A.; Kovalenko, M. V. Nanocrystals of Cesium Lead Halide Perovskites ($CsPbX_3$, X= Cl, Br, and I): Novel Optoelectronic Materials Showing Bright Emission with Wide Color Gamut. *Nano lett.* **2015**, *15*, 3692–3696.

(S4) Swarnkar, A.; Marshall, A. R.; Sanehira, E. M.; Chernomordik, B. D.; Moore, D. T.; Christians, J. A.; Chakrabarti, T.; Luther, J. M. Quantum Dot-Induced Phase Stabilization of α-$CsPbI_3$ Perovskite for High-Efficiency Photovoltaics. *Science* **2016**, *354*, 92–95.

(S5) de Weerd, C.; Gomez, L.; Capretti, A.; Lebrun, D. M.; Matsubara, E.; Lin, J.; Ashida, M.; Spoor, F. C.; Siebbeles, L. D.; Houtepen, A. J., et al. Efficient Carrier Multiplication in $CsPbI_3$ Perovskite Nanocrystals. *Nat. Commun.* **2018**, *9*, 4199.

(S6) Eperon, G. E.; Paternò, G. M.; Sutton, R. J.; Zampetti, A.; Haghighirad, A. A.; Cacialli, F.; Snaith, H. J. Inorganic Caesium Lead Iodide Perovskite Solar Cells. *J. Mater. Chem. A* **2015**, *3*, 19688–19695.

(S7) Ding, X.; Cai, M.; Liu, X.; Ding, Y.; Liu, X.; Wu, Y.; Hayat, T.; Alsaedi, A.; Dai, S. Enhancing the Phase Stability of Inorganic α-$CsPbI_3$ by the Bication-Conjugated Organic Molecule for Efficient Perovskite Solar Cells. *ACS Appl. Mater. Interfaces* **2019**, *11*, 37720–37725, PMID: 31588720.

(S8) Eperon, G. E.; Stranks, S. D.; Menelaou, C.; Johnston, M. B.; Herz, L. M.; Snaith, H. J. Formamidinium of Formamidinium Lead Trihalide: A Broadly Tunable Perovskite for Efficient Planar Heterojunction Solar Cells. *Energy Environ. Sci.* **2014**, *7*, 982.

(S9) Kim, Y. G.; Kim, T.-Y.; Oh, J. H.; Choi, K. S.; Kim, Y.-J.; Kim, S. Y. Cesium Lead Iodide Solar Cells Controlled by Annealing Temperature. *Phys. Chem. Chem. Phys.* **2017**, *19*, 6257–6263.

(S10) Cho, N.-K.; Na, H.-J.; Yoo, J.; Kim, Y. S. Long-Term Stability in γ-$CsPbI_3$ Perovskite via an Ultraviolet-Curable Polymer Network. *Commun. Mater.* **2021**, *2*, 30.

(S11) Zhao, H.; Xu, J.; Zhou, S.; Li, Z.; Zhang, B.; Xia, X.; Liu, X.; Dai, S.; Yao, J. Preparation of Tortuous 3D γ-$CsPbI_3$ Films at Low Temperature by CaI_2 as Dopant for Highly Efficient Perovskite Solar Cells. *Adv. Funct. Mater.* **2019**, *29*, 1808986.

(S12) Duan, L.; Zhang, H.; Liu, M.; Gratzel, M.; Luo, J. Phase-Pure gamma-$CsPbI_3$ for Efficient Inorganic Perovskite Solar Cells. *ACS Energy Lett.* **2022**, *7*, 2911–2918.

(S13) Deretzis, I.; Bongiorno, C.; Mannino, G.; Smecca, E.; Sanzaro, S.; Valastro, S.; Fisicaro, G.; La Magna, A.; Alberti, A. Exploring the Structural Competition between the Black and the Yellow Phase of $CsPbI_3$. *Nanomaterials* **2021**, *11*.

(S14) De Roo, J.; Ibáñez, M.; Geiregat, P.; Nedelcu, G.; Walravens, W.; Maes, J.; Martins, J. C.; Van Driessche, I.; Kovalenko, M. V.; Hens, Z. Highly Dynamic Ligand Binding and Light Absorption Coefficient of Cesium Lead Bromide Perovskite Nanocrystals. *ACS nano* **2016**, *10*, 2071–2081.
